# A genome-wide screening for RNAi pathway proteins in Acari

**DOI:** 10.1186/s12864-020-07162-0

**Published:** 2020-11-12

**Authors:** Beatrice T. Nganso, Noa Sela, Victoria Soroker

**Affiliations:** Institute of Plant Protection, Agricultural Research Organization, the Volcani Center, P.O.B 15159, 7505101 Rishon leZion, Israel

## Abstract

**Background:**

RNA interference (RNAi) is a highly conserved, sequence-specific gene silencing mechanism present in Eukaryotes. Three RNAi pathways are known, namely micro-RNA (miRNA), piwi-interacting RNA (piRNA) and short interfering RNA (siRNA). However, little knowledge exists about the proteins involved in these pathways in Acari. Moreover, variable successes has been obtained in gene knockdown via siRNA pathway in their functional genomics and management. We hypothesized that the clue may be in the variability of the composition and the efficacy of siRNA machinery among Acari.

**Results:**

Both comparative genomic analyses and domain annotation suggest that all the analyzed species have homologs of putative core proteins that mediate cleaving of targeted genes via the three RNAi pathways. We identified putative homologs of *Caenorhabditis elegans* RNA-dependent RNA polymerase (RdRP) protein in all species though no secondary Argonaute homologs that operate with this protein in siRNA amplification mechanism were found, suggesting that the siRNA amplification mechanism present in Acari may be distinct from that described in *C. elegans*. Moreover, the genomes of these species do not encode homologs of *C. elegans* systemic RNAi defective-1 (Sid-1) protein that mediate silencing of the mRNA target throughout the treated organisms suggesting that the phenomena of systemic RNAi that has been reported in some Acari species probably occur through a different mechanism. However, homologs of putative RNAi spreading defective-3 (Rsd-3) protein and scavenger receptors namely Eater and SR-CI that mediate endocytosis cellular update of dsRNA in *C. elegans* and *Drosophila melanogaster* were found in Acari genomes. This result suggests that cellular dsRNA uptake in Acari is endocytosis-dependent. Detailed phylogenetic analyses of core RNAi pathway proteins in the studied species revealed that their evolution is compatible with the proposed monophyletic evolution of this group.

**Conclusions:**

Our analyses have revealed the potential activity of all three pathways in Acari. Still, much experimental work remains to be done to confirm the mechanisms behind these pathways in particular those that govern systemic/parental RNAi and siRNA amplification in Acari. Disclosure of these mechanisms will facilitate the development of new and specific management tools for the harmful species and enrichment of the beneficial species.

**Supplementary Information:**

**Supplementary information** accompanies this paper at 10.1186/s12864-020-07162-0.

## Background

With over 55,000 described species, Acari (mites and ticks) are the most speciose group of organisms within the subphylum Chelicerata, which is considered to be the second most diverse group of terrestrial organisms after the subphylum Hexapoda to which insects belong [1]. This apparently monophyletic group is subdivided into two lineages (super-orders) based on their morphological characteristics, feeding habits and ecological niches namely: the Parasitiformes and Acariformes [2, 3]. While many Acari species provide important ecosystem services, others are known to impose serious economic and health problems to humans, managed crops, wild and domestic animals [2, 4, 5]. Harmful Acari cause direct and indirect damages on their hosts through their feeding activities and transmission of deadly pathogens, respectively. Although, many strategies have been successfully implemented to manage these pests, chemical treatment with synthetic acaricides remains the topmost management option that is currently used [6–8]. The economic losses to global food and animal production due to these harmful Acari and expenditure on acaricide usage to manage them worth billions of dollars [5, 9, 10]. Growing evidence indicates loss of efficacy to these chemicals as mites developed resistance to most of them [8, 11]. Moreover, synthetic acaricides are notorious to the environment and non-target organisms. Therefore, new management options for harmful Acari are required.

RNA interference (RNAi) has been widely explored in the management of harmful Acari as well as in functional genomics [12, 13]. The ability for sequence-specific gene silencing by this molecular tool is known to be highly conserved in eukaryotic organisms [14, 15]. So far, three pathways for RNAi have been described in Eukaryotes, namely: The micro-RNA (miRNA), the piwi-interacting RNA (piRNA) and the short interfering RNA (siRNA) [16–18]. Although these pathways differ in a number of ways, interactions between them are known to occur [17, 19]. Firstly, while siRNA pathway can be exo- and endogenously triggered, the other two pathways are strictly endogenously triggered [17]. Secondly, both siRNA and miRNA pathways are triggered by small interfering RNAs (siRNAs) and microRNAs (miRNAs), respectively, transcribed from double-stranded RNAs (dsRNAs) that depend on the Dicer proteins in the cytoplasm for their biogenesis [17, 19]. In contrast, the piRNA pathway is triggered by piwi-interacting RNAs (piRNAs), transcribed from single-stranded RNA (ssRNA) precursors that are independent of Dicer proteins for their biosynthesis [19, 20]. Thirdly, all the three pathways depend on the proteins of the Argonaute superfamily for silencing the target genes, but the active Argonaute protein family differs between the pathways. Both siRNA and miRNA pathways depend on the Ago family Argonautes whereas the piRNA pathway depends on the Piwi family Argonautes [20–22]. Lastly, these pathways can cross-regulate each other as reported in a few eukaryotic organisms [17].

The experimental application of RNAi for gene silencing in arthropods including Acari exploits the siRNA pathway, which is activated mainly via the exogenous administration of specific synthetic dsRNAs [12, 13, 23–25]. In principle, three molecular processes are expected to define the success of gene silencing: the uptake of dsRNA by the target cell, the amplification of the siRNAs and the transport of the latter to distant cells to induce gene silencing throughout the treated organism, a phenomenon referred to as systemic RNAi [23]. These processes are chains of reactions involving a number of essential proteins. However, information on the number of proteins involved, their structure and function is still scanty in Acari [13, 26].

The first step following the administration of the trigger dsRNA molecule is its cellular uptake, which occurs either via a transmembrane channel-mediated mechanism and/or an endocytosis-mediated mechanism [23]. In the nematode- *Caenorhabditis elegans*, dsRNA uptake within somatic and germline cells involves both mechanisms. The cellular uptake of dsRNAs via the transmembrane channel mechanism is facilitated by a conserved systemic RNAi defective 1 (Sid-1) protein [27, 28]. Meanwhile, the uptake via the endocytosis mechanism is facilitated by the conserved RNAi spreading defective-3 (Rsd-3) protein [29]. In addition, other proteins namely: Rsd-2 and Rsd-6 mediate cellular dsRNA uptake only in *C. elegans* germline cells [30]. In *D. melanogaster*, cellular uptake of dsRNA is mediated predominantly by scavenger receptor endocytosis proteins: Eater and SR-CI [31, 32] and Rsd-3 [33]. The siRNA amplification process has been described in plants, fungi, *C. elegans* and a few other organisms and requires the action of RNA-dependent RNA polymerase (RdRP) [18, 23, 34, 35]. In *C. elegans*, the secondary siRNAs produced by RdRPs from primary siRNAs following Dicer cleavage associate with a specific group of Argonaute proteins of the Wago family to direct target gene cleavage [34, 36, 37]. In *C. elegans*, the Sid-1 protein already mentioned above is involved in systemic RNAi [27, 28]. Homologs of this protein have been reported in other organisms including insects [33], but not yet in Acari. However, apparently the systemic spread is not entirely dependent on this protein, as organisms lacking this gene are still able to show systemic RNAi [33].

Even though successful gene silencing and systemic RNAi have been reported in some Acari species using siRNA pathway, failure or variable success in gene knockdown has also been reported (reviewed in [13, 26]). The reasons for the variable success or failure in gene silencing could be partly due to difference among the species in the proteins that are involved in either of its processes as was suggested for similar problems in insects (reviewed in [38]). Given the complexity and the interactions between the RNAi cascades, siRNA pathway efficacy may be connected to the structure and function of the other two RNAi pathways, which are also rarely described in Acari. In order to shed some light on the possible pathways that may be involved in Acari, we searched for putative homologs of proteins involved in the three RNAi pathways as well as those involved in cellular uptake of dsRNAs, siRNA amplification and systemic RNAi in the genome assemblies of five species of Parasitiformes and four species of Acariformes annotated at the protein levels.

## Results

### Proteins involved in biosynthesis of the small RNAs

The proteins such as Drosha, Pasha, Exportin1, 2 and 5, Loquacious, R2D2 and Dicer are the first in the biosynthesis of miRNAs and siRNAs in eukaryotes [17, 19, 37, 39]. In miRNA pathway, the RNase III enzyme Drosha associates with a dsRNA binding protein, Pasha to generate pre-miRNAs in the nucleus [17, 19]. Subsequently, the pre-miRNAs are exported into the cytoplasm by nuclear export receptors, Exportin1, 2 and/or 5 for further processing by Dicer [37, 39]. Also, Dicer is the first protein that is required in the siRNA biogenesis to cleave long dsRNA precursors into short siRNAs capable of regulating anti-viral responses and transposable elements [17]. In *D. melanogaster* two Dicer proteins are known: Dicer1 partners with a dsRNA binding protein, Loquacious to generate the miRNAs and Dicer2 partners with another dsRNA binding protein, R2D2 to generate siRNAs [40].

Using the orthology detection tool called Proteinortho5 [39] and/or Blastp against the NCBI protein database, we found homologs of Drosha, Pasha, Exportin1 and Exportin2 in the genomes of ecto-parasitic mites: *Varroa destructor*, *V. jacobsoni* and *Tropilaelaps mercedesae*, the predatory mite: *Metaseiulus occidentalis* and the black-legged tick: *Ixodes scapularis*. All of these species belong to Parasitiformes. Proteins similar to Exportin5 were also found in the genomes of these species, except *T. mercedesae*. Using the same search approaches, we also found homologs of all these proteins in Acariformes species that include the dust mites: *Dermatophagoides pteronyssinus*, *Euroglyphus maynei*, the two-spotted spider mite: *Tetranychus urticae* and the scabies mite: *Sarcoptes scabiei*. As presented in Table 1, multiple copies of Drosha, Pasha, Exportin1 and 5 homologs were found only in the genomes of *V. destructor*, *V. jacobsoni*, *T. urticae*, *D. pteronyssinus*, *E. maynei* and *I. scapularis*. Using InterProScan [40], HmmScan [41] and the online motif search tool (https://www.genome.jp/tools/motif/), we further confirmed the presence or absence of known conserved domains in homologs of these proteins identified in all these Acari species (Table 1).

**Table 1:** Number of protein homologs involved in the biosynthesis of miRNAs and their characteristic conserved domains. Asterisk (*) following species name abbreviations indicates that its genome is fully sequenced whereas no asterisk indicates that its genome is partially sequenced. (^#^) Indicates that the protein contains no conserved domains

Proteins	Domain description	Acari Super-order	Parasitiformes					Acariformes			
		Acari orders	Ixodida	Mesostigmata				Trombidiformes	Sarcoptiformes		
		Domain ID (IPR/Pfam)	Is*	Mo*	TM	Vd	Vj	Tu*	Dp	Em	Ss
Drosha	Ribonuclease domain	IPR000999/PF14622	1	1	1	6	3	1	1	1	1
	One DsRNA binding domain	IPR014720/PF00035									
Pasha	DsRNA binding domain	IPR014720/PF00035	2	1	1	3	3	2	2	1	1
Exportin1	Importin-beta_N	IPR001494/PF03810	1	1	1	2	2	3	1	1	1
	Exportin-1/Importin-b-like	IPR013598/PF08389									
	Exportin-1, C-domain	IPR014877/PF08767									
Exportin2	Importin-beta_N	IPR001494/PF03810	1	1	1	1	1	1	1	1	1
	Exportin-2_C	IPR005043/PF03378									
	Exportin-2_Central	IPR013713/PF08506									
Exportin5	Exportin-1/Importin-b-like	IPR013598/PF08389	1	1	0	1	1	2	1^#^	2^#^	1^#^

Proteins shown are from *Ixodes scapularis* (Is), *Metaseiulus occidentalis* (Mo), *Tropilaelaps mercedesae* (Tm), *Tetranychus urticae* (Tu), *Varroa destructor* (Vd), *Varroa jacobsoni* (Vj), *Dermatophagoides pteronyssinus* (Dp) *Euroglyphus maynei* (Em) and *Sarcoptes scabiei* (Ss)

**Fig. 1:**
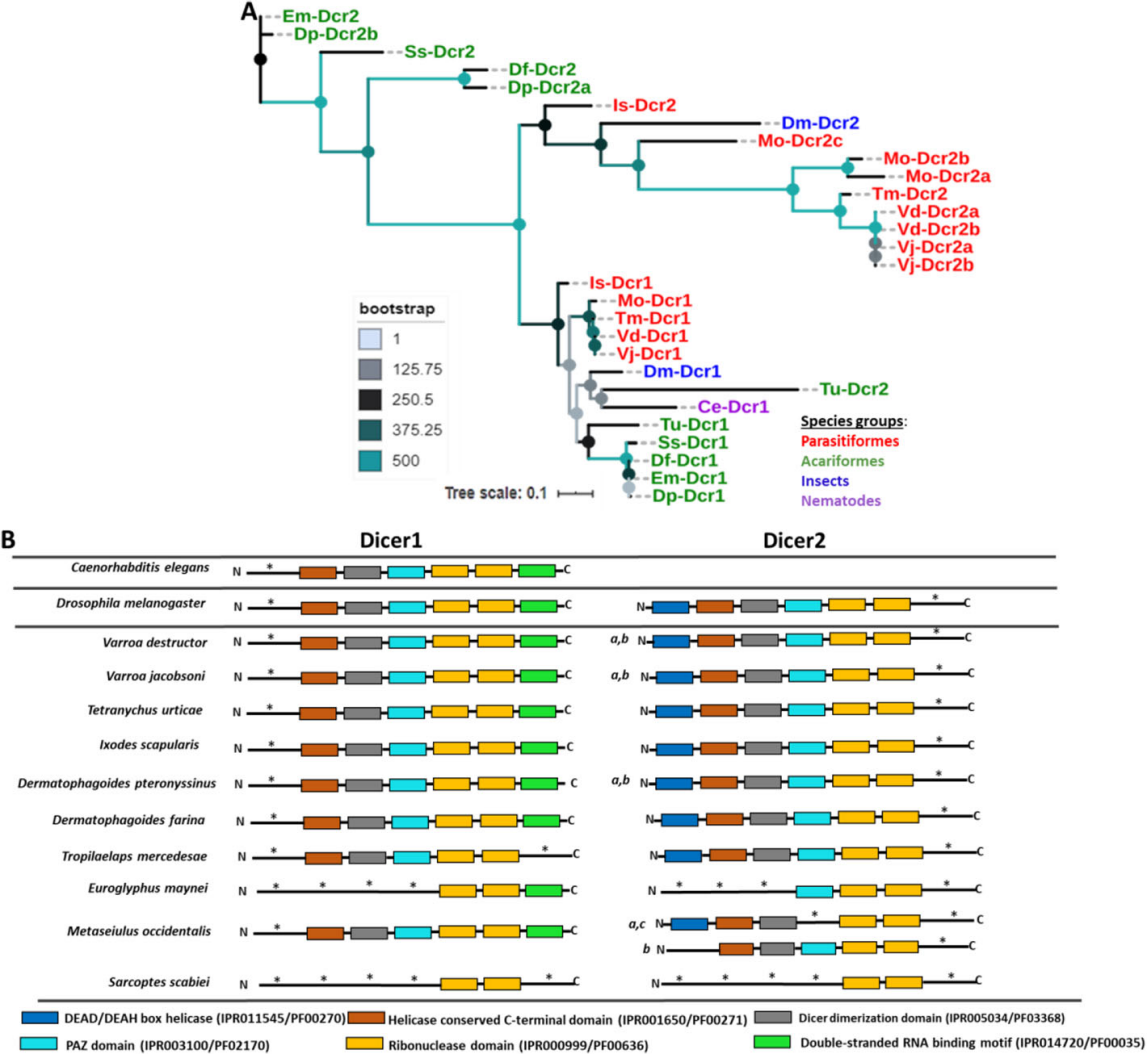
Phylogenetic analysis and schematic domain architecture of Dicer proteins. The tree in **a** was built based on the alignment of the two catalytic conserved Ribonuclease domains (IPR000999/PF00636) of Dicer homologs identified in the studied Acari species using MAFFT [42]. The studied species include: *Varroa destructor* (Vd), *Varroa jacobsoni* (Vj), *Tetranychus urticae* (Tu), *Ixodes scapularis* (Is), *Dermatophagoides pteronyssinus* (Dp), *Tropilaelaps mercedesae* (Tm), *Euroglyphus maynei* (Em), *Metaseiulus occidentalis* (Mo) and *Sarcoptes scabiei* (Ss). Homologs of Dicer proteins in *Caenorhabditis elegans* (Ce), *Drosophila melanogaster* (Dm) and *Dermatophagoides farinae* (Df) were also included in this tree. The species names were abbreviated for convenience and the letters (a, b and c) following the names of some species indicate the number of protein copies found in their genomes. The tree was constructed using PhyML [43], with the model recommended by Lefort et al. [44] under the Akaike information criterion (AIC) (LG + G) with 500 bootstrap replicates. The domain architecture of Dicer proteins in **b** was generated by searching for known conserved domains in the Pfam database using InterProScan [40], HmmScan [41] and the online motif search tool (https://www.genome.jp/tools/motif/). The letters (a, b and c) indicate the number of protein copies found in their genomes

With the same search approaches mentioned above, we identified homologs of Dicer1 (Dcr1) and Dicer2 (Dcr2) proteins in the genome assemblies of all the studied Acari species. The phylogenetic analysis revealed that homologs of Dicer1 and Dicer2 in Acariformes species clustered separately from those found in Parasitiformes species (Fig. 1a). Moreover, we found that Dicer1 homologs identified in *V. destructor* (Vd), *V. jacobsoni* (Vj), *T. urticae* (Tu), *I. scapularis* (Is) and *D. pteronyssinus* (Dp), clustered together with significant bootstrap support (Fig. 1a) and have similar domain architecture to Dicer1 of both *D. melanogaster* (Dm-Dcr1) and *C. elegans* (Ce-Dcr1) (Fig. 1). In the same vein, Dicer2 homologs in these species clustered together and have similar domain architecture to Dm-Dcr2 with the exception of the homolog identified in *T. urticae*, which clustered with Ce-Dcr1 (Fig. 1a, b). A single Dicer1 homolog was found in all these species while several copies of Dicer2 were detected in the genome assemblies of *V. destructor* (2), *V. jacobsoni* (2) and *D. pteronyssinus* (2). As shown in Fig. 1a, duplicates of Dicer2 in *V. destructor* and *V. jacobsoni* clustered together while those identified in *D. pteronyssinus* did not. It is worthwhile to note that duplicates of Dicer2 in *V. destructor*, *V. jacobsoni* and *D. pteronyssinus* have similar domain architecture to Dm-Dcr2 (Fig. 1b).

Dicer1 homolog found in *T. mercedesae* (Tm) clustered with Vj-Dcr1 and Vd-Dcr1 with significant bootstrap values (Fig. 1a) though it did not have similar domain architecture with Dm-Dcr1 and Ce-Dcr1 (Fig. 1). Homolog of Dicer2 in this species has a similar domain structure with Dm-Dcr2 and clustered closely with Vj-Dcr2 and Vd-Dcr2 (Fig. 1a, b). On the other hand, homologs of Dicer1 and Dicer2 identified in *E. maynei* and *S. scabiei* clustered closely with Dp-Dcr1, Df-Dcr1, Dp-Dcr2 and Df-Dcr2 (Fig. 1a) though they differed significantly in domain architecture from Dm-Dcr1/Ce-Dcr1 and Dm-Dcr2, respectively (Fig. 1b). As shown in Fig. 1a and b, one homolog of Dicer1 found in *M. occidentalis* (Mo-Dcr1) clustered with Tm-Dcr1, Vj-Dcr1 and Vd-Dcr1 with significant bootstrap values and clearly shared similarities in domain architecture with Dm-Dcr1 and Ce-Dcr1. However, the three Dicer2 homologs identified in this species (Mo-Dcr2a,b,c) grouped significantly with homologs of this protein identified in *I. scapularis*, *T. mercedesae*, *V. destructor* and *V. jacobsoni* though they did not have similar domain structure with Dm-Dcr2 (Fig. 1a, b).

We also identified homologs of the cofactor of Dicer 1, Loquacious protein, in the genomes of all Acari species investigated herein and several copies of this protein were found only in *V. jacobsoni* (6) and *T. urticae* (2) genomes (see Additional file 1). All homologs of this protein found in these Acari species have the characteristic conserved dsRNA binding domain (IPR014720/PF00035). However, we did not find homologs of the cofactor of Dicer 2, the R2D2 protein and its homolog C3PO protein previously identified in the genome of the red flour beetle *Tribolium castaneum* [33], in the genomes of any of the studied Acari species (see Additional file 1).

### Argonautes and RISC components

Argonautes are the core effector proteins of the RNA-induced silencing complexes (RISCs) in the cytoplasm. They belong to the Argonaute superfamily, which is functionally divided into three families based on their interactions with specific RNA substrates: Ago, Wago and Piwi [19]. Other RISC components, Vasa intronic gene (Vig-1) and Tudor-staphylococcal nuclease (Tsn-1), that are known to contribute to the degradation of the target mRNAs [45, 46], were also identified in the genome of the Acari in this study, with the exception of *E. maynei*, which had Vig-1 but not Tsn-1 homolog (see Additional file 1).

### Ago family Argonautes

**Fig. 2:**
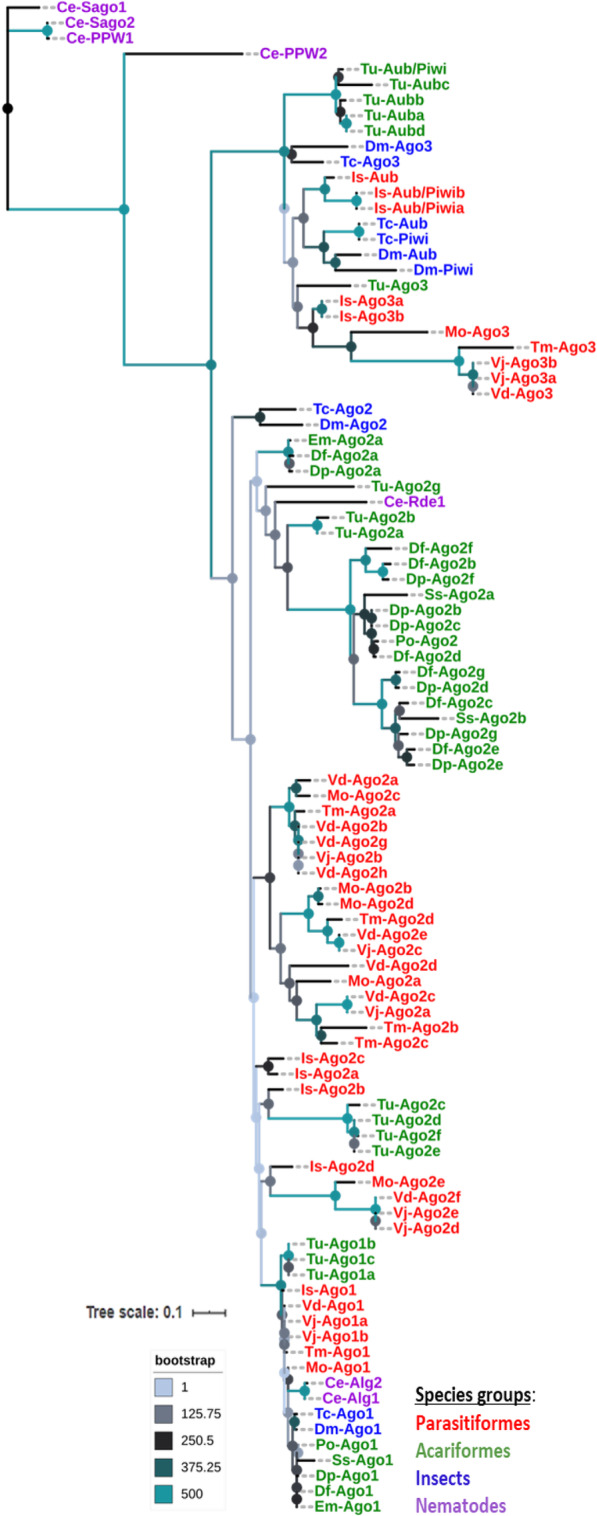
Phylogenetic analysis of Argonaute proteins. The tree was constructed based on the alignments of the conserved PIWI domain of Argonaute homologs identified in the studied Acari species: *Varroa destructor* (Vd), *Varroa jacobsoni* (Vj), *Tetranychus urticae* (Tu), *Ixodes scapularis* (Is), *Dermatophagoides pteronyssinus* (Dp), *Tropilaelaps mercedesae* (Tm), *Euroglyphus maynei* (Em), *Metaseiulus occidentalis* (Mo) and *Sarcoptes scabiei* (Ss) using MAFFT [42]. Homologs of these proteins in *Caenorhabditis elegans* (Ce), *Drosophila melanogaster* (Dm), *Tribolium castaneum* (Tc), *Psoroptes ovis* (Po) and *Dermatophagoides farinae* (Df) were also included in this tree. The species names were abbreviated for convenience and the letters (a, b, c, d, e, f, g and h) following the names of some species indicate the number of protein copies found in their genomes. The tree was constructed using PhyML [43], with the model recommended by Lefort et al. [44] under the Akaike information criterion (AIC) (LG + G) with 500 bootstrap replicates

In this study, we found homologs of Ago family Argonautes in Parasitiformes and Acariformes species using the same search approaches mentioned above. A maximum likelihood tree based on the alignment of the PIWI domain showed that homologs of the Ago family identified in Acari belong to two distinct groups that appear to be specialized in either mi- or siRNA- directed gene silencing (Fig. 2). It was intriguing to find that some of the Acari homologs clustered together with Dm-Ago1, Ce-Alg1 and Ce-Alg2, whereas others clustered closely with Dm-Ago2 and Ce-Rde1. Therefore, we referred to these homologs as Ago1 and Ago2, respectively. Except in *E. maynei* whose genome encodes a single copy of Ago1 and Ago2, we found more copies of Ago2 than Ago1 in the genomes of the remaining Acari species. The tree further showed that homologs of Ago1 and Ago2 in Acariformes species clustered separately from those identified in Parasitiformes species, with significant bootstrap values (Fig. 2).

**Fig. 3:**
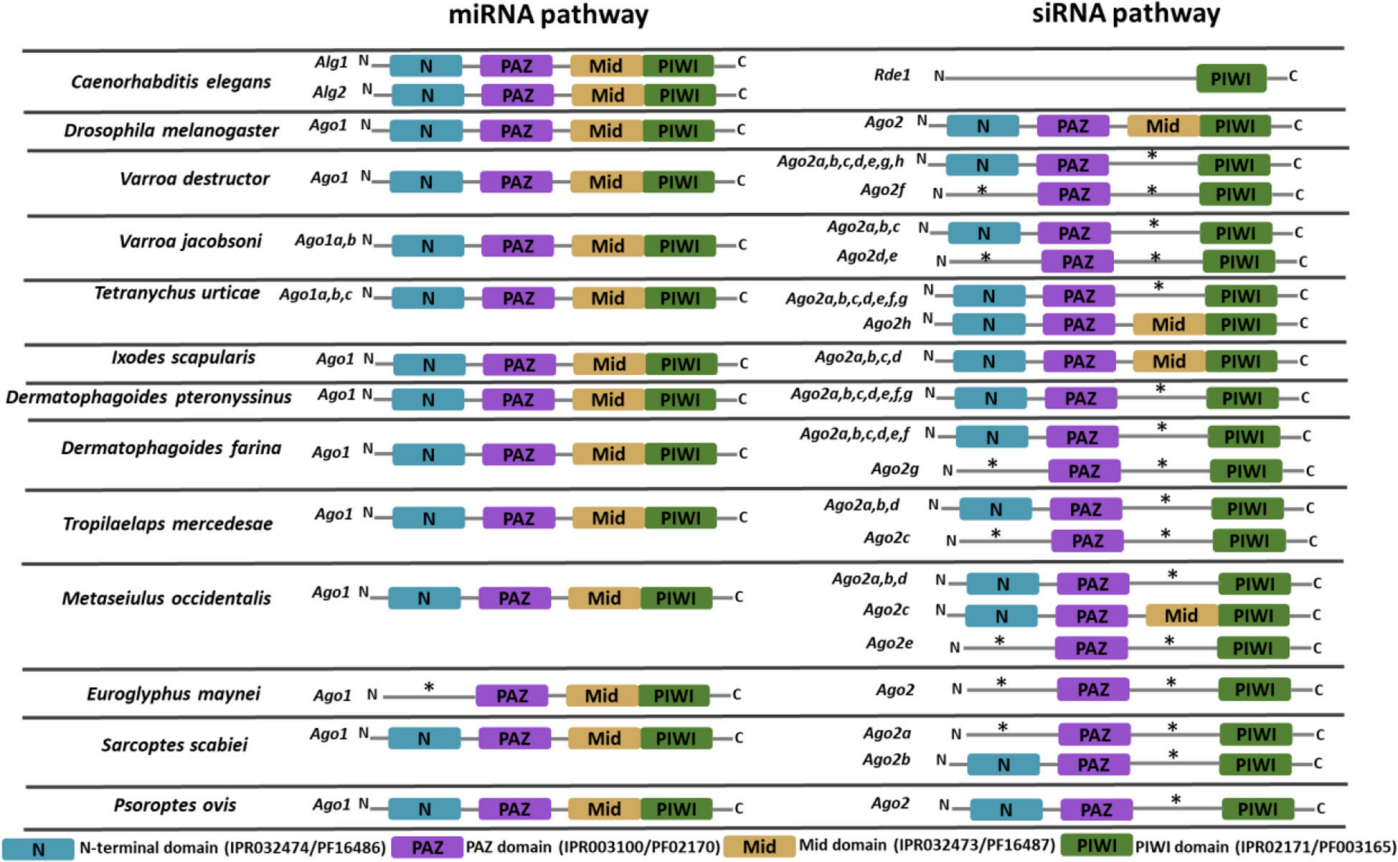
Schematic domain architecture of Argonaute proteins of the Ago family in Acari. The domain architecture of the proteins was generated by searching for known conserved domains in the Pfam database using InterProScan [40], HmmScan [41] and the online motif search tool (https://www.genome.jp/tools/motif/). The letters (a, b, c, d, e, f, g and h) following the names of the protein (Ago1or Ago2) indicate the number of protein copies found in the individual Acari species

The search for the presence of known conserved domains revealed that almost all the homologs of Ago1 in Acari have the same domain architecture with Dm-Ago1 and Ce-Alg1 and 2, with the exception of the single homolog identified in *E. maynei*, which lacks the N-terminal domain (Fig. 3). Contrary to Ago1 homologs identified in Acari, which mostly shared the same domain architecture with homologs of this protein in *C. elegans* and *D. melanogaster*, most of the Ago2 homologs in Acari had a domain architecture that was quiet distinct from those of both *C. elegans* and *D. melanogaster* (Fig. 3). The majority of them lack the Mid domain or both the N-terminal and the Mid domains. All the homologs of Ago-2 identified in *I. scapularis* (Ago-2a, b, c and d) and one homolog of this protein identified in *M. occidentalis* (Ago-2c) and *T. urticae* (Ago-2 h) have similar domain structure with Dm-Ago2.

### Piwi family Argonautes

Argonautes from the Piwi family such as Argonaute-3 (Ago3), Aubergine (Aub) and Piwi were found in the genomes of *I. scapularis, T. urticae*, *V. destructor*, *V. jacobsoni*, *M. occidentalis* and *T. mercedesae*, but not in genomes of *D. pteronyssinus*, *E. maynei* and *Sarcoptes scabiei*. We identified homologs of Ago3 in the genomes of *I. scapularis, T. urticae*, *V. destructor*, *V. jacobsoni*, *M. occidentalis* and *T. mercedesae* (see Additional file 1). Notably, several copies of this homolog were found only in the genomes of *I. scapularis* (2) and *V. jacobsoni* (2). We further identified putative homologs of *D. melanogaster*-Aub and *Tribolium castaneum* (Tc)-Aub/Piwi only in the genome assemblies of *I. scapularis* and *T. urticae* (see Additional file 1). It is important to note that one homolog of Drosophila Aub/Piwi and Ago-3 were previously detected in the genome of the red flour beetle, *Tribolium castaneum* (Tc) [33]. In our study, we identified one and two copies of Dm-Aub and Tc-Aub/Piwi, respectively, in *I. scapularis* and one and four copies of Tc-Aub/Piwi and Dm-Aub, respectively in *T. urticae* (see Additional file 1). All homologs of the Piwi family Argonautes identified in *T. urticae* and *I. scapularis* had the PIWI and PAZ domains, while those identified in *V. destructor*, *V. jacobsoni*, *M. occidentalis* and *T. mercedesae* had only the PIWI domain. Phylogenetic analysis placed homologs of Ago3, Aub and Piwi in Acari with homologs of these proteins in *D. melanogaster* and *T. castaneum* (Fig. 2).

### Wago family Argonautes

We found homologs of the Wago family Argonautes (Ce-PPW1, −PPW2, −Sago1 and -Sago2) in some Acari species studied herein using Proteinortho5 [39] (see Additional file 1). One and two homologs of Sago2 and Sago1, respectively, were identified in *I. scapularis*’s genome. Also, one homolog of PPW2 was found in *T. mercedesae* while one and three homologs of Sago1 were identified in *V. jacobsoni* and *V. destructor*, respectively. In *T. urticae*, homologs of Sago1 (1) and PPW2 (1) were also found. Interestingly, most of the homologs of the Wago family Argonautes were highly similar to Ago family Argonautes (Ago2) identified in these species (represented as Is-Ago2a and Ago2b, Tm- Ago2c, Vd-Ago2b, Vj-Ago2b and Tu-Ago2c) (see Additional file 1). Since all the homologs of the Wago family Argonautes clustered with those of the Ago family Argonautes, with significant bootstrap support (Fig. 2), we tentatively referred to them as members of the Ago family Argonautes that are Ago2 homologs. It is worth mentioning that all *C. elegans* Wago family Argonautes used as query sequences in our study have both the PAZ and the PIWI domains.

### Catalytic residues of ago and Piwi families Argonautes

Homologs of the Ago and Piwi families Argonautes identified in all these Acari species were further examined for the presence of the conserved catalytic residues, Aspartate-Aspartate-Histidine/Aspartate/Lysine (DDH/DDD/DDK). These residues that are present within the catalytic PIWI domain presumably enable some Argonaute proteins to cleave the target mRNAs (reviewed in [47, 48]). As shown on Table 2, the DDH catalytic residue was found in all the Argonautes identified in *I. scapularis*. Similarly, Argonautes of the Ago family identified in *E. maynei*’s genome contained this motif. The only member of the Piwi family that did not have any of these residues in the sequences of their PIWI domains, is the Ago3 identified in the genomes of *M. occidentalis*, *T. mercedesae* and *V. jacobsoni* whereas most Argonautes of the Ago family identified in these species have the DDH residue. Also, Ago3 and one homolog of Ago-2 (that is Ago2d) identified in *V. destructor* lacked these conserved residues, though the remaining proteins of the Ago family had the conserved DDH residue. Also, Ago1 of *S. scabiei* did not have these residues, whereas Ago2 homologs did have. In *T. urticae*, all the Argonautes of the Ago family had the DDH motif, while only four out of the six Argonautes of the Piwi family had the DDH motif. In *D. pteronyssinus*, Ago1 and Ago2a have the DDH motif, while the remaining Argonautes that were Ago2b to 2 g had the DDD motif.

**Table 2:**
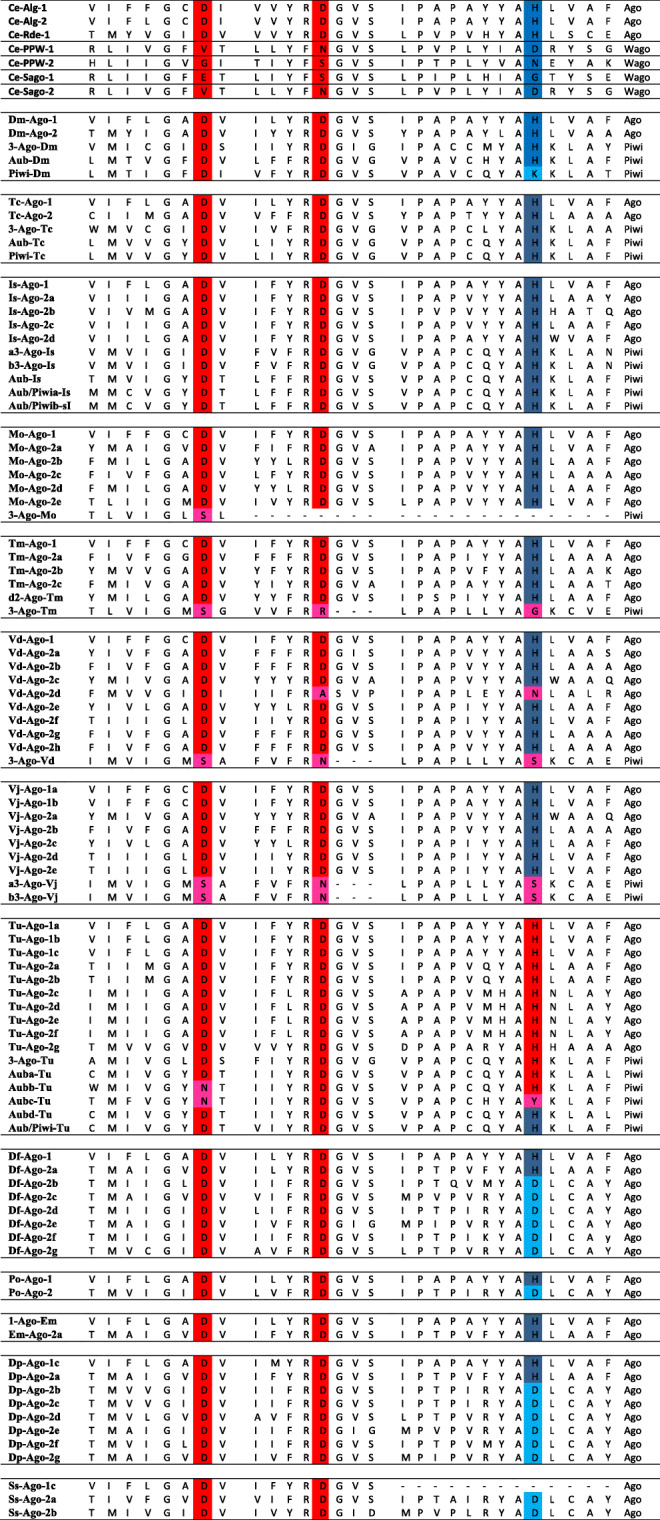
Catalytic residues of Argonautes of the Ago, Piwi and Wago groups in Acari species, *Caenorhabditis elegans*, *Drosophila melanogaster* and *Tribolium castaneum*

Argonautes shown are from *Caenorhabditis elegans* (Ce), *Drosophila melanogaster* (Dm), *Tribolium Castaneum* (Tc), Ixodes Scapularis (Is), *Metaseiulus occidentalis* (Mo), *Tropilaelaps mercedesae* (Tm), *Varroa destructor* (Vd), *Varroa jacobsoni* (Vj), *Tetranychus urticae* (Tu), *Dermatophagoides farinae* (Df), Psoroptes ovis (Po), *Euroglyphus maynei* (Em), *Dermatophagoides pteronyssinus* (Dp), and *Sarcoptes scabiei* (Ss). The amino acid sequences of the PIWI domains were aligned with MAFFT [42]. Substitutions for the Aspartate-Aspartate-Histidine (Asp-Asp-His) motif such as Aspartate or Histidine to either Serine (S), Arginine (R), Alanine (A), Asparagine (N), Glycine (G) or Tyrosine (Y), are colored in pink. Substitutions of the Histidine to Aspartate (D) or Lysine (K) are colored in light blue. (−) indicate the absence of amino acid residues from the sequence

### DsRNA uptake and spread

We searched for Sid-1, Rsd-2, Rsd-3, Rsd-6, Eater and SR-CI proteins that are involved in cellular dsRNA uptake and systemic spread of the silencing dsRNA molecules as mentioned above. We were unable to identify proteins similar to Sid-1 nor its homologs SilA, SilB and SilC, previously found in the *T. castaneum* genome [33] in the studied Acari species. Similarly, homologs of Rsd-2 and Rsd-6 were not found in any of the studied Acari species, though homologs of Rsd-3 protein were present in all of them (see Additional file 1). Duplicates of this protein were only found in the genomes of *I. scapularis* (2), *V. jacobsoni* (5) and *V. destructor* (9) respectively. Interestingly, all the identified homologs had a single conserved domain, the epsin N-terminal homology (ENTH) domain that has been shown to be sufficient to mediate the transport of dsRNA molecules into both somatic and germ cells of *C. elegans* [29]. We also identified homologs of Dm-Eater in the genomes of some Acari species but not in those of *D. pteronyssinus*, *E. maynei* and *S. scabiei*. Again, duplicates of this protein were found in *T. urticae* (3), *V. destructor* (5) and *V. jacobsoni* (2). Homologs of this protein in the Acari species have several epidermal growth factor (EGF)-like modules. Furthermore, one homolog of Dm-SR-CI receptor was identified only in the genome of *T. urticae*. Alike the Drosophila SR-CI receptor, its homolog had a characteristic conserved domains coding for Meprin, A5 protein, and tyrosine phosphatase Mu (MAM).

### siRNA secondary amplification

**Fig. 4:**
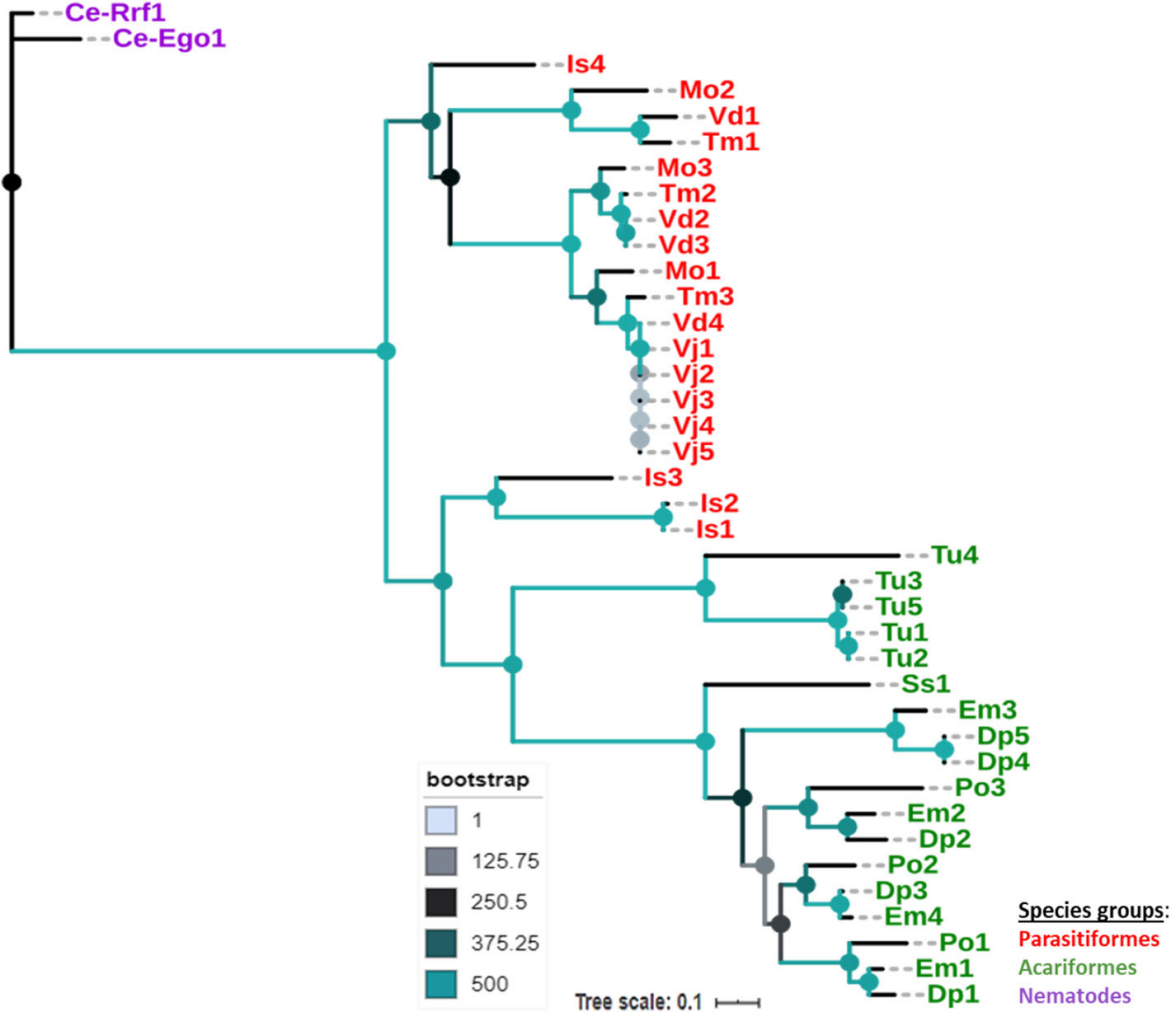
Phylogenetic distribution of RNA-dependent RNA polymerase (RdRP) proteins in Acari species: *Varroa destructor* (Vd), *V. jacobsoni* (Vj), *Tropilaelaps mercedesae* (Tm), *Metaseiulus occidentalis* (Mo), *Ixodes scapularis* (Is), *Dermatophagoides pteronyssinus* (Dp), *Euroglyphus maynei* (Em), *Tetranychus urticae* (Tu), *Sarcoptes scabiei* (Sc) and *Psoroptes ovis* (Po). The nematode species, *Caenorhabditis elegans* (Ce) was used as outgroup. The species names were abbreviated for convenience and the numbers (1, 2, 3, 4 and 5) following their names indicate the protein copies found in each species. The tree was constructed using PhyML [43], with the model recommended by Lefort et al. [44] under the Akaike information criterion (AIC) (LG + G + I + F) with 500 bootstrap replicates and is based on the amino acid alignment of the conserved RdRP domain (IPR007855/PF05183) using MAFFT [42]

Systemic and trans-generational gene interference rely on the amplification of the initial siRNA trigger for efficient gene knockdown throughout the treated organism. This mechanism for enhancing RNAi potency necessitates the action of a cellular RNA-dependent RNA polymerase (RdRP) protein, which has been extensively studied in *C. elegans* [49, 50]. Interestingly, we found that all Acari species from the two sister lineages, Acariformes and Parasitiformes studied here, had such RdRP proteins. Duplicates of this protein were found in the genomes of all studied species. Moreover, all the Acari RdRP proteins identified in this study including those previously identified in *C. elegans* (Ego-1 and Rrf-1) [37] and other Acari species e. g. *D. farinae* [51] and *Psoroptes ovis* [52] had the characteristic conserved RdRP domain [34]. In order to infer the evolutionary history of Acari RdRP proteins, we conducted a phylogenetic analysis based on the amino acid alignment of RdRP domain using *C. elegans* as outgroup, as described in the methods section. The phylogeny revealed that proteins of Acariformes (indicated with “green” color on Fig. 4) clustered strongly together (494/500 bootstraps) and separately from those of Parasitiformes. The Parasitormes proteins (indicated with “red” color on Fig. 4) also clustered strongly together (408/500 bootstraps). However, three out of the total four proteins identified in *I. scapularis* were found to be evolutionary closer to Acariformes proteins than to those of Parasitiformes (479/500 bootstraps) (Fig. 4).

## Discussion

### Proteins associated with the three RNAi pathways in Acari

Our findings demonstrate that the genomes of all the nine species that belong to diverse Acari orders of agricultural, veterinary and medical importance encode homologs of the proteins that mediate the silencing process in the three RNAi pathways. In particular, we demonstrate that the genomes of all these species encode homologs of the RNase III enzyme Drosha, its co-factor Pasha and nuclear export receptors, Exportin1, 2 and/or 5 (Tables 1 and 3) thereby suggesting that these proteins, which play vital roles in the initial step of miRNA biogenesis inside the nucleus are conserved in Acari as in other animal species [37, 53–56].

**Table 3:** Summary of the numbers of protein orthologs associated with the three RNAi pathways identified in the nine investigated Acari genomes. Asterisk (*) following species name abbreviation indicates that its genome is fully sequenced whereas no asterisk indicates that its genome is partially sequenced

Proteins	Acari Super-order	Parasitiformes					Acariformes			
	Acari orders	Ixodida	Mesostigmata				Trombidiformes	Sarcoptiformes		
	Species/Pathway	Is*	Mo*	TM	Vd	Vj	Tu*	Dp	Em	Ss
Drosha	miRNA	1	1	1	6	3	1	1	1	1
Dicer1	miRNA	1	1	1	1	1	1	1	1	1
Dicer2	siRNA	1	3	1	2	2	1	2	1	0
Argonaute1	miRNA	1	1	1	1	2	3	1	1	1
Argonaute 2	siRNA	4	5	4	8	5	8	7	1	2
Argonaute 3	piRNA	2	1	1	1	2	1	0	0	0
Aubergine/Piwi	piRNA	3	0	0	0	0	5	0	0	0
Rsd-3	Cellular dsRNA uptake	2	1	1	9	5	1	1	1	1
Eater		1	1	1	5	2	3	0	0	0
SR-CI		0	0	0	0	0	1	1	1	1
RdRP	siRNA amplification	4	3	3	4	5	5	5	4	1
Sid-1/SilA/SilB/SilC	Systemic RNAi	0	0	0	0	0	0	0	0	0

Proteins shown are from *Ixodes scapularis* (Is), *Metaseiulus occidentalis* (Mo), *Tropilaelaps mercedesae* (Tm), *Varroa destructor* (Vd), *Varroa jacobsoni* (Vj), *Tetranychus urticae* (Tu), *Dermatophagoides pteronyssinus* (Dp) *Euroglyphus maynei* (Em) and *Sarcoptes scabiei* (Ss)

Functional specialization of Dicer and Argonaute proteins is apparent in Acari. It is supported by the phylogenetic analysis of Dicer proteins which showed two Dicer paralogs with distinct functions in Acari: Dicer1 and Dicer2 involved in miRNA and siRNA biosynthesis, respectively (Table 3, Fig. 1a, b) as was previously shown in the other two Acari species: *P. ovis* [52] and *D. farina* [51]. Two Dicer paralogs were also found in insect species [33, 57–59] and crustaceans [56, 60] analyzed so far, but only one (Dicer1) was so far detected in nematodes and mammals [19]. In case of Argonautes, our phylogenetic analysis revealed representatives of two families (Ago and Piwi) in Acari (Table 3, Fig. 2) out of the three families known in eukaryotes [17, 19]. Within the Ago family, we further detected representatives of two distinct functional groups: Ago1 and Ago2 specialized in miRNA- and siRNA-directed gene silencing, respectively. The fact that these two gene families clustered separately from Wago family Argonaute specific to *C. elegans* with significant bootstrap value indicates that Acari, as insects [33] and crustaceans [24, 54], lack clear orthologs of Wago family Argonautes in their genomes. The match that occurred between *C. elegans* Wago family Argonautes and some orthologs of Ago family Argonautes (Ago2) in Acari, supports the idea of a common ancestor for both protein families as was suggested by Swarts et al. [61]. The finding that functional separation of core effector proteins involved in the exogenous siRNA and endogenous miRNA/piRNA machineries have occurred in Acari as in insects [33, 59] and crustaceans [24, 54, 56], supports the previous hypothesis that duplication and specialization of Dicers and Ago family Argonautes have occurred already in the ancestral Arthropoda [58], early before its split into two monophyletic groups: Chelicerata and Mandibulata, back in the Precambrian era [62]. However, further analysis of homologs of these proteins in the other Arthropod lineages is needed to ascertain whether these phenomena are widely conserved or merely lineage-specific.

This functional specialization of the effector RNAi proteins may suggest that there is no competition for these proteins among the RNAi cascades in Acari and probably in insects as well. On the other hand, the pathways may be interlinked. Is it possible that the exogenous activation of the siRNA pathway via dsRNA treatment will affect the endogenous regulatory RNAi machineries (miRNA/piRNA/siRNA), which in turn may have a positive or negative effect on the silencing efficiency of the target gene in Acari? A recent study conducted in the pea aphid, *Aphis pisum* (Hemiptera: Aphididae) showed that the activation of the exogenous siRNA pathway following dsRNA treatment led to a significant change in the expression levels of both the exogenous siRNA and endogenous miRNA/piRNA associated genes thereby suggesting that crosstalk among these pathways can occur [63]. In fact, crosstalk among these three RNAi pathways have already been reported in a few eukaryotic species [17]. In addition, the Loquacious gene, identified in the Acari species is known to be required for both the endogenous siRNA and miRNA machineries in *D. melanogaster* [64], thus indicating that these pathways may compete for this protein [17]. Therefore, future studies to understand the interconnection that exists among these RNAi pathways in Acari, may expedite the efficient application of RNAi to both functional genomics and pest management.

Phylogenetic analysis of Dicer and Argonaute proteins in Acari revealed a high divergence of exogenous siRNA-associated Dicer2 and Ago2 proteins, but not of endogenous miRNA-associated Dicer1 and Ago1 proteins and piRNA-associated Piwi Argonaute, thereby suggesting a strong selective pressure on the exogenous siRNA core proteins. For example, the genomes of *V. destructor*, *V. jacobsoni*, *M. occidentalis* and *D. pteronyssinus* even encode for more than one copy of Dicer2 (Table 3). The genomes of all the studied Acari species, except for *E. maynei*, encode for more than one copy of Ago2 (Table 3). This high divergence suggests that the ancestral role of exogenous siRNA machinery in antiviral immunity is conserved in Acari [22]. Among the species investigated, it was intriguing to find that species such as *V. destructor* and *T. urticae* known to be intimately associated with several viruses [65–67] had the highest number of copies of these proteins, especially Ago2 (Table 3). This may suggest that these two species evolved several copies of these proteins as a mechanism to counteract the negative effects of viruses that are associated with them. It is important to note that *T. urticae* is one of the most serious polyphagous arthropod pest with more than 1000 host plant species identified to date [5, 9, 10] while *V. destructor* of the family Varroidae and its associated viruses is the most important ecto-parasite that is responsible for honey bee (*Apis mellifera*) colony losses almost worldwide [65–67]. The ancestral siRNA-based antiviral defense is also maintained in insects [18], crustaceans [24, 56] and plants [68, 69] but not in vertebrates [70, 71]. this may have occurred because of evolution of an interferon system regarded as the primer route of defense against viruses in vertebrates rather than on RNAi-based antiviral defense system [70, 71].

### Slicing activities of Dicer and Argonaute proteins

The phylogenetic analysis of Dicer proteins based on the alignment of the two Ribonuclease slicing domains showed that their homologs clustered according to their functional relatedness to either siRNA or miRNA pathway with the exception of Dicer2 homolog identified in *T. urticae*, which clustered closely to Dicer1 of *C. elegans* (Fig. 1b). Could it be that a Dicer2 homolog in *T. urticae* may be involved in both pathways? Since very little is known about the slicing activity of Dicer proteins in Acari, we cannot resolve this question yet, The analysis of the ATPase hydrolitic activity of the conserved Helicase domain of Dicer1 and Dicer2 in *T. urticae* may provide an insight into their cleavage preference for either miRNA or siRNA substrates. It has been reported in other organisms that the processing of RNA substrates by some Dicer homologs is influenced by its Helicase domain [72–74].

For most of the miRNA- and siRNA-class Argonautes in Acari, we found the highly conserved DDH or DDD catalytic motif in their PIWI domains, which may suggest their slicing abilities. However, the presence of this motif may not be sufficient for slicing the target mRNA transcripts (reviewed in [46]). On the other hand, the majority of the homologs of the Piwi family Argonautes identified in *V. destructor*, *V. jacobsoni*, *T. mercedesae*, *M. occidentalis*, *I. scapulari* and *T.urticae* lack the conserved DDH/DDD/DDK motif in their PIWI domains. This motif was substituted by Serine-Arginine-Glycine (SRG), Serine-Asparagine-Serine (SRS) and/or Asparagine-Aspartate-Histidine/Tyrosine (NDH/NDY) (Table 2). With these substitutions, it remains unclear whether the catalytic activity of these proteins is still intact.

### Endocytosis mechanism for dsRNA uptake and systemic RNAi in Acari

Our findings suggest that cellular uptake of dsRNA in Acari is strongly endocytosis-dependent. In fact, the genomes of all the Acari species contained a clear homolog of Rsd-3 protein (Table 3), which is an integral component of systemic RNAi in *C. elegans.* In this organism, it facilitates the importation of dsRNAs in both somatic and germ cells via endocytosis [29]. In some Acari like *I. scapularis*, *V. jacobsoni* and *V. destructor*, we even found more than one copy of this protein (Table 3). Marr and colleagues also found a copy of this protein in the *P. ovis* genome [52]. Additionally, proteins similar to scavenger receptors Eater and SR-CI that mediate endocytosis-dependent dsRNA uptake in S2 cells of *D. melanogaster* [31, 32] were found in some Acari species (Table 3). Also, previous study demonstrated by RNAi approach, that receptor-mediated endocytosis cellular dsRNA uptake occurs in the tick *Haemaphysalis longicornis* [75]. Overall, these studies suggest that this is a general phenomenon in Acari. The fact that endocytosis-mediated cellular dsRNA uptake mechanism is present in nematodes, insects, crustaceans and Acari genomes [33, 76] may suggest that this mechanism evolved very early in metazoan evolution.

Regarding the systemic transfer, our study revealed that the Sid-1 protein that mediates systemic RNAi in *C. elegans* somatic and germ cells [27, 28] or its homologs found in *T. castaneum* [33] are both absent from the genomes of all the studied Acari species (Table 3), including that of *P. ovis* genome [52]. The apparent absence of Sid-1 protein in Acari may imply that systemic RNAi reported in *M. occidentalis* [77], *T. urticae* [78] and *I. scapularis* [79] occurred via a different mechanism. In fact, in insects, systemic RNAi can occur independently of this protein [33].

### siRNA amplification mechanism in Acari

The potency of RNAi gene silencing throughout the organism depends on an amplification mechanism mediated by an RNA-dependent RNA polymerase protein as mentioned above. We confirmed the presence of homologs of this protein along with their characteristic conserved domain in the genomes of all the Acari species (Table 3) as was commonly found in other arthropods including insects, crustaceans, chelicerates and myriapods [35]. We also confirmed that duplications of this protein has occurred in almost all the studied species, except in *S. scabiei* (see Additional file 1). Previous studies also reported the presence of several copies of this protein in the genomes of *P. ovis* [52] and *D. farina* [51]. Overall, this and previous genomic studies indicate that RdRP amplification activity seems to occur in Acari species studied to date, which may result in efficient gene silencing throughout the organisms as was suggested for arthropod species in which this protein was found [80]. In fact, systemic and trans-generational gene silencing have been reported in *M. occidentalis* [77], *T. urticae* [78] and *I. scapularis* [79]. Interestingly, our analysis of Argonaute proteins did not reveal any clear homologs of Wago Argonautes, described in the model organism *C. elegans*, in any of the Acari species. This finding supports the recent suggestion of Pinzon and colleagues that the role of RdRP proteins in siRNA amplification is not conserved and probably varies among different animal species [35]. It remains to be determined, what is the siRNA amplification mechanism in Acari.

## Conclusions

Using genomic data annotated at the protein level, we provide for the first time information on the putative proteins in nine Acari species that maybe involved in the gene silencing processes of the three RNAi pathways in both Acariformes and Parasitiformes lineages. It is clear that Acari, like insects and crustaceans have evolved a set of specialized proteins for gene silencing in the three RNA pathways and an endocytosis mechanism for cellular dsRNA uptake. However, the mechanisms and molecular basis through which siRNA amplification and systemic/parental RNAi occur in Acari, still remain a puzzle and require follow up by structural and functional studies. A complete annotation of genome sequences of the Acari species studied herein as well as additional studies from other families and more closely related Acari lineages (e.g. other Arachnids) might not only shed light into the underlying mechanisms and molecular basis of siRNA amplification and systemic RNAi, but no doubt also uncover additional RNAi pathway proteins. So far, our results suggest that all the evaluated Acari species have a potential for active exogenous siRNA-directed gene silencing machinery, though difference in gene knockdown is likely to occur given that the number of copies of core RNAi proteins vary among the species. Our findings provide a basis for exploiting siRNA pathways in functional genomics and in the management of economically important species, especially species like *T. mercedesae*, *D. pteronyssinus* and *E. maynei* in which RNAi-gene silencing has not yet been exploited. Moreover, additional studies are needed to elucidate the regulatory mechanisms behind RNAi pathways as well as any possible cross talk mechanisms. Our phylogenetic analyses of the core RNAi proteins: Dicer, Argonaute and RdRP revealed that homologs of these proteins grouped according to their respective orders and those identified in Acariformes species clustered separately from those found in Parasitiformes species. This finding thus provides additional support for this taxonomic division in the subclass Acari [3]. Still transcriptomic, proteomic as well as functional studies are needed to confirm the exact existence and function of the putative proteins identified in this study before these findings can be implemented in any solutions for pest management.

## Methods

### Homolog identification of core RNAi pathway proteins

**Fig. 5:**
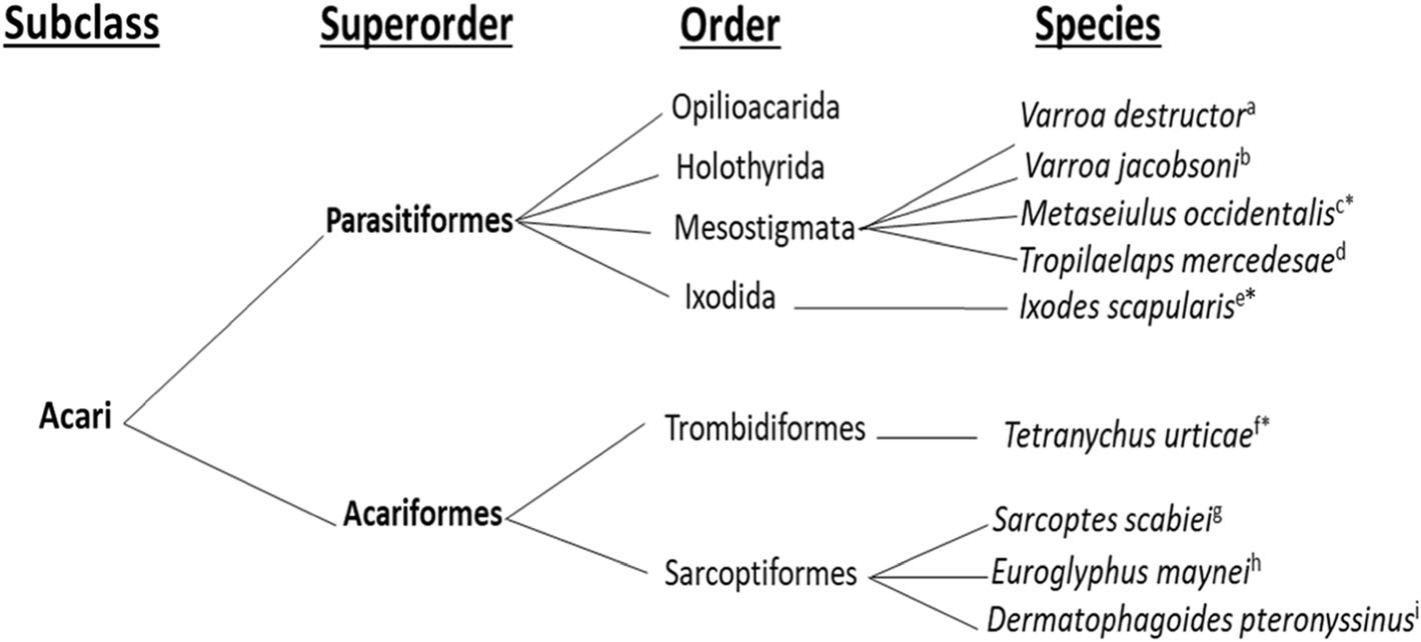
Phylogenetic relationship of the nine Acari species investigated in this study. This classification is adapted from [3], with modifications. Asterick (*) indicates species with fully annotated genomes whereas no Asterick indicates species with a partial annotated genomes. ^a^From Cornman et al. [81], ^b^From Techer et al. [82], ^c^From Hoy et al. [83], ^d^From Dong et al. [84], ^e^From Miller et al. [85], ^f^From Grbić et al. [86], ^g^From Rider et al. [87], ^h^From Rider et al. [88], iFrom Waldron et al. [89]

In this study, we selected five and four species of Parasitiformes and Acariformes, respectively, whose genomes have been annotated at the protein level. Within the superorder Parasitiformes, *V. destructor* (RefSeq assembly accession: GCF_002443255.1), *V. jacobsoni* (GCF_002532875.1), *M. occidentalis* (GCF_000255335.1) and *T. mercedesae* (GenBank assembly accession: GCA_002081605.1) belonging to order Mesostigmata and *I. scapularis* (GCF_002892825.2) belonging to the order Ixodida were chosen in our study (Fig. 5). Within the superorder Acariformes, *T. urticae* (GCF_000239435.1) found in the order Trombidiformes and *S. scabiei* (GCA_000828355.1), *E. maynei* (GCA_002135145.1) and *D. pteronyssinus* (GCF_001901225.1) found in the order Sarcoptiformes were also selected. Within the superorder Parasitiformes, species of the orders Opilioacarida and Holothyrida were not selected in this study because they lack either a genome sequence or an annotated genome at the protein level. The Benchmarking Universal Single-Copy Orthologs (BUSCO, version 3.0.2) strategy was used according to Simão et al. [90] to assess the quality of the individual genome assemblies used in this study as shown in the Additional file 2.

In the genome assembly of an individual organism, we searched for RNAi pathway proteins belonging to the following functional groups: small RNA biosynthesis proteins, Argonautes and RISC components, siRNA amplification protein and dsRNA uptake and spread proteins as summarized in the Additional file 1 using Proteinortho5 (https://www.bioinf.uni-leipzig.de/Software/proteinortho/) [39] and/or Blastp search against the NCBI database. As query sequences, we used homologous proteins within the genomes of *P. ovis*, *D. farinae*, *D. melanogaster*, *T. castaneum* and *C. elegans*. The Amino acid sequences of the query proteins for the first four species were retrieved from the NCBI database, while those for the fifth organism were retrieved from the WormBase database (shown in the Additional file 2). The accession numbers of the query sequences are available in the Additional files 1 and 2. Only homologs that yielded top blastp hits were retained. We further verified these hits by Blastp search in NCBI and annotated them as potential RNAi proteins only when the Blastp search provided the query sequence as the best hit. The direct web links of genome assemblies, annotations and accession numbers of protein sequences corresponding to all the studied Acari species obtained from the NCBI database are shown in the Additional files 1 and 2.

### Domain annotation of protein sequences

The putative gene homologs identified in this study were thereafter classified and annotated on the basis of their known conserved domains retrieved from the Pfam database using InterProScan (https://www.ebi.ac.uk/interpro/search/sequence/) [40], HmmScan (https://www.ebi.ac.uk/Tools/hmmer/search/hmmscan) [41] and the online motif search tool (https://www.genome.jp/tools/motif/) with default settings. They were defined as follows: DEAD/DEAH helicase, PAZ, two Ribonuclease, Helicase C terminal, Dicer dimerization and dsRBD domains for Dicers; two Ribonuclease and dsRBD domains for Drosha; N-terminal, PAZ, Mid and PIWI domains for Argonautes; dsRBD for Pasha and loquacious; RdRP domain for RdRP; Importin-beta_N, Exportin-1/Importin-b-like and Exportin-1, C-domain for Exportin-1; Importin-beta_N, Exportin-2_C and Exportin-2_Central for Exportin-2; and Exportin-1/Importin-b-like for Exportin-5.

### Phylogenetic analyses of Dicer, Argonaute and RNA-dependent RNA polymerase proteins

Protein or conserved domain sequences were aligned using MAFFT version 7.452 [42]. Erroneous regions that were poorly aligned and/or contained many gaps were curated with Gblocks version 0.91b [91]. The realigned sequences were then used for the construction of phylogenetic trees using the maximum likelihood (ML) methods with 500 bootstraps in PhyML version 3.0 [43]. The generated phylogenetic trees were thereafter visualized using the interactive Tree Of Life (iTOL) server [92].

Phylogenetic trees of homologs for Dicer protein identified in the Acari species that were based on the alignment of full-length Dicer protein and the two conserved Ribonuclease domains were constructed using additional sequences from *C. elegans* (Dicer1:K12H4.8), *D. melanogaster* (Dicer1: NP_524453.1, Dicer2: NP_523778.2) and *D. farina* (Dicer1: AUI38412.1, Dicer2: AUI38413.1). The tree for homologs of Argonaute proteins that was based on the alignment of the conserved PIWI domain was constructed using additional sequences from *C. elegans* (Alg1: F48F7.1, Alg2: T07D3.7, Rde-1: K08H10.7, PPW1:C18E3.7, PPW2: Y110A7A.18, Sago1: K12B6.1, Sago2: F56A6.1), *D. melanogaster* (Ago1: NP_725341.1, Ago2: ABB54719.1, Ago3: ABO27430.1, Aub: AGA18946.1, Piwi: AAD08705.1), *T. castaneum* (Ago1: EFA09197.2, Ago2: EFA11590.1, Ago3: EFA02921.1, Aub: XP_008196303.1, Piwi: EFA07425.1), *P. ovis* (Ago1: SZF06500.1, Ago2: SZF06480.1) and *D. farina* (Ago1: AUI38415.1, Ago2a: AUI38416.1, Ago2b: AUI38417.1, Ago2c: AUI38418.1, Ago2d: AUI38419.1, Ago2e: AUI38420.1, Ago2f: AUI38421.1, Ago2g: AUI38422.1). The tree for homologs of RdRP protein that was based on the alignment of the conserved RdRP domain was constructed using additional sequences from *C. elegans* (Ego1: F26A3.3 and Rrf1: F26A3.8). We only considered domains which confercatalytic properties to the given protein (Dicer, Argonaute and RdRP enzymes). The amino acid sequences of the conserved domains of query proteins retrieved from the Pfam database and all the accession numbers of protein sequences used in the phylogenetic analyses of Dicer, Argonautes and RdRP proteins are shown in the Additional file 2.

## Supplementary Information

**Additional file 1.** Orthologs of RNAi pathway proteins identified in the genome assembly of Metaseiulus occidentalis (Mo) by Proteinortho5 and/or Blastp against NCBI protein database (GCF_000255335.1). Orthologs of RNAi pathway proteins identified in the genome assembly of Varroa destructor (Vd) by Proteinortho5 and/or Blastp against NCBI protein database (GCF_002443255.1). Orthologs of RNAi pathway proteins identified in the genome assembly of Varroa jacobsoni (Vj) by Proteinortho5 and/or Blastp against NCBI protein database (GCF_002532875.1). Orthologs of RNAi pathway proteins identified in the genome assembly of Ixodes scapularis (Is) by Proteinortho5 and/or Blastp against NCBI protein database (GCF_002892825.2). Orthologs of RNAi pathway proteins identified in the genome assembly of Tropilaelaps mercedesae (Tm) by Proteinortho5 and/or Blastp against NCBI protein database (GCA_002081605.1). Orthologs of RNAi pathway proteins identified in the genome assembly of Tetranychus urticae (Tu) by Proteinortho5 and/or Blastp against NCBI protein database (GCF_000239435.1). Orthologs of RNAi pathway proteins identified in the genome assembly of Dermatophagoides pteronyssinus (Dp) by Blastp against NCBI protein database (GCF_001901225.1). Orthologs of RNAi pathway proteins identified in the genome assembly of Euroglyphus maynei (Em) by Blastp against NCBI protein database (GCA_002135145.1). Orthologs of RNAi pathway proteins identified in the genome assembly of Sarcoptes scabiei (Ss) by Blastp against NCBI protein database (GCA_000828355.1).

**Additional file 2: Table S1.** Quality evaluation of the genome assemblies investigated in this study. **Table S2.** Accession numbers of Dicer protein sequences used in phylogenetic tree construction. **Table S3.** Accession numbers of Argonaute protein sequences used in phylogenetic tree construction. **Table S4.** Accession numbers of RNA-dependent RNA polymerase (RdRP) protein sequences used in phylogenetic tree construction.

## Data Availability

All data generated or analyzed during this study are included in this published article (Table 1) and its additional files (Additional files 1 and 2).

## Abbreviations

AIC: Akaike information criterion
BUSCO: Benchmarking Universal Single-Copy Orthologs
dsRNA: double stranded RNA
iTOL: Interactive Tree Of Life
miRNA: micro-RNA
piRNA: piwi-interacting RNA
RdRP: RNA-dependent RNA polymerase
RISC: RNA-induced silencing complex
RNAi: RNA interference
Rsd-3: RNAi spreading defective-3
Sid-1: Systemic RNAi defective-1
siRNA: short interfering RNA
ssRNA: single stranded RNA
Tsn-1: Tudor-staphylococcal nuclease-1
Vig-1: Vasa intronic gene-1
