# Supplementary material for: A genome-wide screening for RNAi pathway proteins in Acari
Source: BMC Genomics. 2020 Nov 12;21:791. doi: 10.1186/s12864-020-07162-0 (PMC7659050; doi:10.1186/s12864-020-07162-0)
Supplement: Supplementary file 2 — Additional file 2: Table S1. Quality evaluation of the genome assemblies investigated in this study. Table S2. Accession numbers of Dicer protein sequences used in phylogenetic tree construction. Table S3. Accession numbers of Argonaute protein sequences used in phylogenetic tree construction. Table S4. Accession numbers of RNA-dependent RNA polymerase (RdRP) protein sequences used in phylogenetic tree construction. [file 12864_2020_7162_MOESM2_ESM.docx]

**Table S1:** Quality evaluation of the genome assemblies investigated in this study

| **Acari lineages and species** | **Assembly methods** | **Links to download genome assemblies, annotations and accession numbers of protein sequences of the studied Acari species obtained from the NCBI database** | **Genome assembly size (Mbp)** | **Nº of protein-coding genes** | **Completeness of the genome according to BUSCO version 3.0.2** |
| --- | --- | --- | --- | --- | --- |
| **Parasitiformes** |  |  |  |  |  |
| *Tetranychus urticae* | GCF_000239435.1 | <https://www.ncbi.nlm.nih.gov/genome/2710?genome_assembly_id=34074> | 90 | 18,414 | 95.1% |
| *Dermatophagoides pteronyssinus* | GCF_001901225.1 | <https://www.ncbi.nlm.nih.gov/genome/8901?genome_assembly_id=322054> | 71 | 5622 | 96.2% |
| *Euroglyphus maynei* | GCA_002135145.1 | <https://www.ncbi.nlm.nih.gov/genome/54478?genome_assembly_id=317541> | 59 | 15,000 | 75.6% |
| *Sarcoptes scabiei* | GCA_000828355.1 | <https://www.ncbi.nlm.nih.gov/genome/36095?genome_assembly_id=219592> | 56 | 10,644 | 95.4% |
|  |  |  |  |  |  |
| **Acariformes** |  |  |  |  |  |
| *Varroa destructor* | GCF_002443255.1 | <https://www.ncbi.nlm.nih.gov/genome/937?genome_assembly_id=335323> | 369 | 11,432 | 97.7% |
| *Varroa jacobsoni* | GCF_002532875.1 | <https://www.ncbi.nlm.nih.gov/genome/62339?genome_assembly_id=342322> | 366 | 15,486 | 98.2% |
| *Tropilaelaps mercedesae* | GCA_002081605.1 | <https://www.ncbi.nlm.nih.gov/genome/53919?genome_assembly_id=313451> | 353 | 15,190 | 95.8% |
| *Metaseiulus occidentalis* | GCF_000255335.1 | <https://www.ncbi.nlm.nih.gov/genome/3487?genome_assembly_id=34535> | 152 | 18,338 | 98.8% |
| *Ixodes scapularis* | GCF_002892825.2 | <https://www.ncbi.nlm.nih.gov/genome/523?genome_assembly_id=408485> | 2081 | 20,486 | 95.2% |

**Table S2:** Accession numbers of Dicer protein sequences used in phylogenetic tree construction.

| **Species Name/Group** | **Abbreviation of Dicer 1 (Dcr1)/**  **Dicer 2 (Dcr2) proteins used in tree construction** | **Accession numbers** | **Database** |
| --- | --- | --- | --- |
| ***Metaseiulus occidentalis* (Mo)**  **(Acari)** | Mo-Dcr1 | XP_018495704.1 | NCBI |
|  | Mo-Dcr2a | XP_028968733.1 | NCBI |
|  | Mo-Dcr2b | XP_028968740.1 | NCBI |
|  | Mo-Dcr2c | XP_028968970.1 | NCBI |
|  |  |  |  |
| ***Varroa destructor* (Vd)**  **(Acari)** | Vd-Dcr1 | XP_022665643.1 | NCBI |
|  | Vd-Dcr2a | XP_022645213.1 | NCBI |
|  | Vd-Dcr2b | XP_022645209.1 | NCBI |
|  |  |  |  |
| ***Varroa jacobsoni* (Vj)**  **(Acari)** | Vj-Dcr1 | XP_022704558.1 | NCBI |
|  | Vj-Dcr2a | XP_022708791.1 | NCBI |
|  | Vj-Dcr2b | XP_022708787.1 | NCBI |
|  |  |  |  |
| ***Ixodes scapularis* (Is)**  **(Acari)** | Is-Dcr1 | XP_029830052.1 | NCBI |
|  | Is-Dcr2 | XP_029830051.1 | NCBI |
|  |  |  |  |
| ***Tropilaelaps mercedesae* (Tm)**  **(Acari)** | Tm-Dcr1 | OQR80281.1 | NCBI |
|  | Tm-Dcr2 | OQR67288.1 | NCBI |
|  |  |  |  |
| ***Tetranychus urticae* (Tu)**  **(Acari)** | Tu-Dcr1 | XP_015789823.1 | NCBI |
|  | Tu-Dcr2 | XP_015784164.1 | NCBI |
|  |  |  |  |
| ***Dermatophagoides pteronyssinus* (Dp)**  **(Acari)** | Dp-Dcr1 | XP_027197063.1 | NCBI |
|  | Dp-Dcr2a | XP_027197670.1 | NCBI |
|  | Dp-Dcr2b | XP_027198147.1 | NCBI |
|  |  |  |  |
| ***Euroglyphus maynei* (Em)**  **(Acari)** | Em-Dcr1 | OTF77775.1 | NCBI |
|  | Em-Dcr2 | OTF81624.1 | NCBI |
|  |  |  |  |
| ***Sarcoptes scabiei* (Ss)**  **(Acari)** | Ss-Dcr1 | KPM03314.1 | NCBI |
|  | Ss-Dcr2 | KPM06069.1 | NCBI |
|  |  |  |  |
| ***Dermatophagoides farinae* (Df)**  **(Acari)** | Df-Dcr1 | AUI38412.1* | NCBI |
|  | Df-Dcr2 | AUI38413.1* | NCBI |
|  |  |  |  |
| ***Drosophila melanogaster* (Dm)**  **(Insecta)** | Dm-Dcr1 | NP_524453.1* | NCBI |
|  | Dm-Dcr2 | NP_523778.2* | NCBI |
|  |  |  |  |
| ***Caenorhabditis elegans* (Ce)**  **(Nematoda)** | Ce- Dcr1 | K12H4.8 | WormBase |

*- The amino acid sequences of the query proteins are presented below and those of the two consecutive Ribonuclease domains (IPR000999/Pf00636) retrieved from the Pfam database appear in red within the sequence of the query proteins.

> Df-Dcr1-AUI38412.1

MLVKYFNPHLSPSDTDSKTLKKIFFLAKSPSSIKLYSSVFNAHCNLRIGEYLETEDAKQW
SCDIWRDKLQSYDIHLMMDDLFEYLIEQELINAKDLNLLILNDVHKILLCTSSTDDCYTR
IIRRLRSQTNEKQYRILGLSASILLEDVSSNVFEKMIEQIENNLGCSCETYADLRMISKY
SIQCRIKLRCYPSLLASKEKSDIIHDETKYRMALFMIRNYSGQFFNFITNVSLDGGITTE
QLINHETIAKMIVDILYIFGTLGEWCALKLIQMVNRELYDTILLLAKTRSNYLRLLNAAH
STLCLIRKSILSYMDAGIVAKLETSSMTSESESRLTLSQFLSISAPKLHLLAHVLFEYFE
ELTSPSTHQTSHSSFTFPSNICSLIYVENRSMALVLDEWLRELVAITRDVNSGKISILEF
LLPDHVFLAEDDGKNELKNFYYRKHQKSLHEIFYYRQQEETLRRFRLAQQCNLLITTSMS
AEGLDVNRCNMVICFDPPKTFHQFIQSKGRVRVECGQFLVLVEKSDHKEYVEKFLQFCNI
ERIFTKLVPLNNQVFIENEPSKFDLMALASMHFIKPPQSKPSLPVSMIHKPRKFDKQINQ
SVNATLQLTLENAISILNRYCNKLPSDTFTKLVPNYKIEIVHSQAESSENSNEVVPKYRC
RLYLPINSTYRDEIMGEIQPTQSLAKQSVAFEAVKTLREIGELDQNFYPVGKETSRYIEK
LGLQDCFISVPKGNQVLQQQHHHSHGPYHQKGNRYNRIQNRNIASKRRQYYNKKVADSIR
GNIFRHDESTNHYYFQYLYEFQMKLTYRLSEEHNTRGRRIIDPAETTRTFGIISPNSLPT
ICDFTIYNRSGEITISIVPIIITDSENGYEITDEKRQQIENFHQYTFENVLPLGKSSLRF
DRQNGSNGNYLIVPINFNAEQDNQKTIDWEFLELIWHHKNDPKSYDQGILRQDDENKFIF
DEQLYRDAVVIPKYRKDKLQAFYYVAEICHYLTPQSPFPDHEYQTFEKYYNQKYGKQITN
LQQPLLDVDHTSARLNLLTPRFLNRRGIHLSSIKSSDHQSKRNPQQKQILVPELCFIHPF
PASFWRKAVCLPCILYRLNLLLIAEELRFKIAKEAKIGVVELQQEKKWPKLDFGWSVLVE
QSRQQTKIEHEQNQFDPIVSNEKQKVISVSNPDDDFIIDTFDPSMAPPPSNDLFTTIENQ
NCSSYPAETINNNFINYDSCSSDIIPQIEIISGPFGYSDKSYSSQRPILPENFMDDDFEE
ESAIVEVDNENNPIVEPAKTLPVRAGSPTYWEDQENFDSNIKSKFGPNFDWRVYESDGND
DDSNSDEFKTSNTEFRFDFEKFCEDIQHYSKMPGLADDIQDNERDFDECDDSRCTENMEM
SSSKRKSTKTNNRKQKPMITSLSDLDDDDESDEQIDDGSYSDFDLNDSSDDYEEDELVDS
NFDDEDSFDDDEDDDYDDEGGDDDNFTNRYSRCKNRKTKTAMDLKIQNLTQTMDAYQTKS
VIYDILKIELLYDSRQIEQKSLKYYYRHRFSLAEQHTEHLRKRKIFDEMYYDEQNQVLQC
VARIQNKQMADSKHESDSVFCDPEYDLIENDKNLNHMNLNQPLVSVLMKKIDTSLLEMMK
IKHEIEDQKIDQQVQNRSQFVNFPEKNSSSTKIEVKFGELLPNRDENLTEFTSNDKNPID
LFQVQRQLALQTLPRWLQMENTSDDQLQIGPHPSLILQAITMSNSSDGINLERLETVGDS
FLKFVKYIMNQINSLLTIFYKALENVEQESSDNVKTTSNKVPYNLLTQHSIPNKSIADCV
EALIGTYLISSGSQGAIQFMDWLGLKVLPKGMKIITKNLPYESSAENKDESYQKHERWLP
QPRSPLIIPKHLRTDAAKVKEIEDKLQENYFKHHLDRFEKIIGYQFRDRAYLVQAFTHNS
YYENTVTDCYQRLEFLGDAVLDYLITRYLYEDSRCHSPGTLTDLRSALVNNTFFAALAVK
YNFHKYLMMLSSELYRVIDGFVRKFNIYYNQNVANGESKKILSAISDFQQGNEDEHMDEN
SYSNYNYWELFVSENEAEHLEDIEVPKALGDIFESVAGAIYLDSGMSLEAVWKAYYPMMK
PEIENFSEKVPKSPIRVLLEKQPQSVKFGKPEINSGRRIRVTVEVFGLGKFVGIGRNKRI
AKCTAAKRALRALESEKRRKELERQRRLQDEFDNFSH

>Df-Dcr2- AUI38413.1

MFKQTIGPYPSEAKRTFFLAPNRVLITQQHKIAETLLPGKITLITGDHNPDNFSSNDWEK
CLKDYQIFFLTPAILYDLLVKGYIKINNINLIVFDEMHWATKKKGRDSGHFYNLIMKLYE
ASNVDDDKKPKILGMSATLIPHKPKEIETFNNSLKKIEQLYHSRVMTKNFERHINHCQEI
VIVYKNENNFNIEIDFDFDEICEKYLNHCFYRNADQMAINGDLNNDESSNGSNDDSMIQE
KDSLKQFRLAFRKIEEYLYKTMGPWFAIEVIKLYDQSLQSMSFEFTELIRRLMNHIRVLI
IKRIEKKFAIENTDDLKLLTDHRLVSDRIRTLLILFDQFRTKTKLTGIIFVKERTTANVL
YRWLLQLSKSNENYSFIKPGFICGATSKDFIKFDQPFHYSQYLSLMKSNLINLIVATSAI
EEGFDIPICNVVIRYDEPQTYRAYSQSRGRARDKNSAYFLLIPQDLRTQMIKMFADFHDM
DNKTKKLKNNVQESLEEEEEDDDEIESDCLDVSDSIIKNMGNFKNGPSTLYPLNSLKLVN
EYLMKLPRDLFSDHFLPMYQFVKDDNDDNNNNDQMKKYAYVFVFPVNSSLSGQLIPGGYF
CTQRAAKIHCAYRTCIHLIQQNEIGDRLNIIDHKFLIKKHHEDLNIRLSVQELQTTDYKK
KFSNFSNQLLLPPSSSSGETADFYHLYALEFDLLTEIDDETDSIGQNEQYFKPKFVGTHN
SSVGILIKNQQKPFKELCSYFFFNLFPIKFRLIPCGKVSIIDTKFELIKHFHQYHAFDLI
LERFRSGRFETSALNTAIMIVLVKRKPYSKQYIIDFDQMEKCRQWIMMMKNSHKDHQSIS
SDYQRNITVYDPSTLDGEFELYGYLCHKKIELLFKKTNEIRLPTDVMNPGESDTTFQQYF
SDKHKRILRHIDQSMVHSKIFRRFYDSFRLIRSIKMTTEKKKTFYNFLPRELLCIYPLSS
ILCLRIMAIPLIMYRLEQFSLSVEFIQPLCQYGIDVDHCDYHVNYHGQKQFESNMKTLKL
EQLAMIDPDSMDKDVPRPKTIGHQNQITNMLQRFLPENESSAIDSNSSSCSENSLKKLFE
ELFSKIKSFLSHTKNKHKSADHHGRVIAQFNPHQETIDYGEVSIFKFEGNIHETIAKEAI
FFDTFDIIQCPNPWQILYGLTLAKAQDLWNIERYETVGDAFIKMTTSLYLYWQYPQYDEY
RLTALKMALISNHNLSLIALEKGIQKFIFGSLNEQIHELFFKRISFMHKDDDDDYLIELK
SKDLADCIEALIGVFLIHGSASTALAFLEYIDLKAFDPRKAKIDLEKQIRQNETVIITDV
HDYNKKSVEEIPLPEIRAKLFDFNPTKPQNYNVFESFYRESLLNDVEEIIDYKFNNKYYL
CKIVVAFTHATHDRSEMLHSILIPSYQQLEFCGDAILDHLVTMYLFVRDEQLGPHEISDI
RSALVNNMFYAHIMVKFQLYKYIRHSSVAITDSITKFVNKFRQNSVGTLLELISGGQNEF
HSQDIDEIDVPKVLSDVFEALVAAIYLDCEFKLDIVWQVVYRMIIRETHFLKNRPKMPLS
RLYNEYPIHSFSDVTTIVQSTKMQLLNQSEMIVTLTVEPYGTFDGRGCNRRQCRTSAAKK
FFTHLRKINVKQTTTSDENM

>Dm-Dcr1-NP_524453.1

MAFHWCDNNLHTTVFTPRDFQVELLATAYERNTIICLGHRSSKEFIALKLLQELSRRARR
HGRVSVYLSCEVGTSTEPCSIYTMLTHLTDLRVWQEQPDMQIPFDHCWTDYHVSILRPEG
FLYLLETRELLLSSVELIVLEDCHDSAVYQRIRPLFENHIMPAPPADRPRILGLAGPLHS
AGCELQQLSAMLATLEQSVLCQIETASDIVTVLRYCSRPHEYIVQCAPFEMDELSLVLAD
VLNTHKSFLLDHRYDPYEIYGTDQFMDELKDIPDPKVDPLNVINSLLVVLHEMGPWCTQR
AAHHFYQCNEKLKVKTPHERHYLLYCLVSTALIQLYSLCEHAFHRHLGSGSDSRQTIERY
SSPKVRRLLQTLRCFKPEEVHTQADGLRRMRHQVDQADFNRLSHTLESKCRMVDQMDQPP
TETRALVATLEQILHTTEDRQTNRSAARVTPTPTPAHAKPKPSSGANTAQPRTRRRVYTR
RHHRDHNDGSDTLCALIYCNQNHTARVLFELLAEISRRDPDLKFLRCQYTTDRVADPTTE
PKEAELEHRRQEEVLKRFRMHDCNVLIGTSVLEEGIDVPKCNLVVRWDPPTTYRSYVQCK
GRARAAPAYHVILVAPSYKSPTVGSVQLTDRSHRYICATGDTTEADSDSDDSAMPNSSGS
DPYTFGTARGTVKILNPEVFSKQPPTACDIKLQEIQDELPAAAQLDTSNSSDEAVSMSNT
SPSESSTEQKSRRFQCELSSLTEPEDTSDTTAEIDTAHSLASTTKDLVHQMAQYREIEQM
LLSKCANTEPPEQEQSEAERFSACLAAYRPKPHLLTGASVDLGSAIALVNKYCARLPSDT
FTKLTALWRCTRNERAGVTLFQYTLRLPINSPLKHDIVGLPMPTQTLARRLAALQACVEL
HRIGELDDQLQPIGKEGFRALEPDWECFELEPEDEQIVQLSDEPRPGTTKRRQYYYKRIA
SEFCDCRPVAGAPCYLYFIQLTLQCPIPEEQNTRGRKIYPPEDAQQGFGILTTKRIPKLS
AFSIFTRSGEVKVSLELAKERVILTSEQIVCINGFLNYTFTNVLRLQKFLMLFDPDSTEN
CVFIVPTVKAPAGGKHIDWQFLELIQANGNTMPRAVPDEERQAQPFDPQRFQDAVVMPWY
RNQDQPQYFYVAEICPHLSPLSCFPGDNYRTFKHYYLVKYGLTIQNTSQPLLDVDHTSAR
LNFLTPRYVNRKGVALPTSSEETKRAKRENLEQKQILVPELCTVHPFPASLWRTAVCLPC
ILYRINGLLLADDIRKQVSADLGLGRQQIEDEDFEWPMLDFGWSLSEVLKKSRESKQKES
LKDDTINGKDLADVEKKPTSEETQLDKDSKDDKVEKSAIELIIEGEEKLQEADDFIEIGT
WSNDMADDIASFNQEDDDEDDAFHLPVLPANVKFCDQQTRYGSPTFWDVSNGESGFKGPK
SSQNKQGGKGKAKGPAKPTFNYYDSDNSLGSSYDDDDNAGPLNYMHHNYSSDDDDVADDI
DAGRIAFTSKNEAETIETAQEVEKRQKQLSIIQATNANERQYQQTKNLLIGFNFKHEDQK
EPATIRYEESIAKLKTEIESGGMLVPHDQQLVLKRSDAAEAQVAKVSMMELLKQLLPYVN
EDVLAKKLGDRRELLLSDLVELNADWVARHEQETYNVMGCGDSFDNYNDHHRLNLDEKQL
KLQYERIEIEPPTSTKAITSAILPAGFSFDRQPDLVGHPGPSPSIILQALTMSNANDGIN
LERLETIGDSFLKYAITTYLYITYENVHEGKLSHLRSKQVANLNLYRLGRRKRLGEYMIA
TKFEPHDNWLPPCYYVPKELEKALIEAKIPTHHWKLADLLDIKNLSSVQICEMVREKADA
LGLEQNGGAQNGQLDDSNDSCNDFSCFIPYNLVSQHSIPDKSIADCVEALIGAYLIECGP
RGALLFMAWLGVRVLPITRQLDGGNQEQRIPGSTKPNAENVVTVYGAWPTPRSPLLHFAP
NATEELDQLLSGFEEFEESLGYKFRDRSYLLQAMTHASYTPNRLTDCYQRLEFLGDAVLD
YLITRHLYEDPRQHSPGALTDLRSALVNNTIFASLAVRHGFHKFFRHLSPGLNDVIDRFV
RIQQENGHCISEEYYLLSEEECDDAEDVEVPKALGDVFESIAGAIFLDSNMSLDVVWHVY
SNMMSPEIEQFSNSVPKSPIRELLELEPETAKFGKPEKLADGRRVRVTVDVFCKGTFRGI
GRNYRIAKCTAAKCALRQLKKQGLIAKKD

>Dm-Dcr2-NP_523778.2

MEDVEIKPRGYQLRLVDHLTKSNGIVYLPTGSGKTFVAILVLKRFSQDFDKPIESGGKRA
LFMCNTVELARQQAMAVRRCTNFKVGFYVGEQGVDDWTRGMWSDEIKKNQVLVGTAQVFL
DMVTQTYVALSSLSVVIIDECHHGTGHHPFREFMRLFTIANQTKLPRVVGLTGVLIKGNE
ITNVATKLKELEITYRGNIITVSDTKEMENVMLYATKPTEVMVSFPHQEQVLTVTRLISA
EIEKFYVSLDLMNIGVQPIRRSKSLQCLRDPSKKSFVKQLFNDFLYQMKEYGIYAASIAI
ISLIVEFDIKRRQAETLSVKLMHRTALTLCEKIRHLLVQKLQDMTYDDDDDNVNTEEVIM
NFSTPKVQRFLMSLKVSFADKDPKDICCLVFVERRYTCKCIYGLLLNYIQSTPELRNVLT
PQFMVGRNNISPDFESVLERKWQKSAIQQFRDGNANLMICSSVLEEGIDVQACNHVFILD
PVKTFNMYVQSKGRARTTEAKFVLFTADKEREKTIQQIYQYRKAHNDIAEYLKDRVLEKT
EPELYEIKGHFQDDIDPFTNENGAVLLPNNALAILHRYCQTIPTDAFGFVIPWFHVLQED
ERDRIFGVSAKGKHVISINMPVNCMLRDTIYSDPMDNVKTAKISAAFKACKVLYSLGELN
ERFVPKTLKERVASIADVHFEHWNKYGDSVTATVNKADKSKDRTYKTECPLEFYDALPRV
GEICYAYEIFLEPQFESCEYTEHMYLNLQTPRNYAILLRNKLPRLAEMPLFSNQGKLHVR
VANAPLEVIIQNSEQLELLHQFHGMVFRDILKIWHPFFVLDRRSKENSYLVVPLILGAGE
QKCFDWELMTNFRRLPQSHGSNVQQREQQPAPRPEDFEGKIVTQWYANYDKPMLVTKVHR
ELTPLSYMEKNQQDKTYYEFTMSKYGNRIGDVVHKDKFMIEVRDLTEQLTFYVHNRGKFN
AKSKAKMKVILIPELCFNFNFPGDLWLKLIFLPSILNRMYFLLHAEALRKRFNTYLNLHL
LPFNGTDYMPRPLEIDYSLKRNVDPLGNVIPTEDIEEPKSLLEPMPTKSIEASVANLEIT
EFENPWQKYMEPVDLSRNLLSTYPVELDYYYHFSVGNVCEMNEMDFEDKEYWAKNQFHMP
TGNIYGNRTPAKTNANVPALMPSKPTVRGKVKPLLILQKTVSKEHITPAEQGEFLAAITA
SSAADVFDMERLEILGDSFLKLSATLYLASKYSDWNEGTLTEVKSKLVSNRNLLFCLIDA
DIPKTLNTIQFTPRYTWLPPGISLPHNVLALWRENPEFAKIIGPHNLRDLALGDEESLVK
GNCSDINYNRFVEGCRANGQSFYAGADFSSEVNFCVGLVTIPNKVIADTLEALLGVIVKN
YGLQHAFKMLEYFKICRADIDKPLTQLLNLELGGKKMRANVNTTEIDGFLINHYYLEKNL
GYTFKDRRYLLQALTHPSYPTNRITGSYQELEFIGDAILDFLISAYIFENNTKMNPGALT
DLRSALVNNTTLACICVRHRLHFFILAENAKLSEIISKFVNFQESQGHRVTNYVRILLEE
ADVQPTPLDLDDELDMTELPHANKCISQEAEKGVPPKGEFNMSTNVDVPKALGDVLEALI
AAVYLDCRDLQRTWEVIFNLFEPELQEFTRKVPINHIRQLVEHKHAKPVFSSPIVEGETV
MVSCQFTCMEKTIKVYGFGSNKDQAKLSAAKHALQQLSKCDA

**Table S3:** Accession numbers of Argonaute protein sequences used in phylogenetic tree construction.

| **Species Name** | **Abbreviation of Argonaute proteins used in tree construction** | **Accession numbers** | **Argonaute group** | **Database** |
| --- | --- | --- | --- | --- |
| ***Metaseiulus occidentalis* (Mo)** | Mo-Ago1 | XP_018496698.1 | Ago | NCBI |
|  | Mo-Ago2a | XP_028967651.1 | Ago | NCBI |
|  | Mo-Ago2b | XP_028968542.1 | Ago | NCBI |
|  | Mo-Ago2c | XP_003741568.1 | Ago | NCBI |
|  | Mo-Ago2d | XP_028968806.1 | Ago | NCBI |
|  | Mo-Ago2e | XP_003747857.1 | Ago | NCBI |
|  | Mo-Ago3 | XP_003742657.2 | Piwi | NCBI |
|  |  |  |  |  |
| ***Varroa destructor* (Vd)** | Vd-Ago1 | XP_022655006.1 | Ago | NCBI |
|  | Vd-Ago2a | XP_022665357.1 | Ago | NCBI |
|  | Vd-Ago2b | XP_022656418.1 | Ago | NCBI |
|  | Vd-Ago2c | XP_022671411.1 | Ago | NCBI |
|  | Vd-Ago2d | XP_022650384.1 | Ago | NCBI |
|  | Vd-Ago2e | XP_022646042.1 | Ago | NCBI |
|  | Vd-Ago2f | XP_022672750.1 | Ago | NCBI |
|  | Vd-Ago2g | XP_022656426.1 | Ago | NCBI |
|  | Vd-Ago2h | XP_022656437.1 | Ago | NCBI |
|  | Vd-Ago3 | XP_022669446.1 | Piwi | NCBI |
|  |  |  |  |  |
| ***Varroa jacobsoni* (Vj)** | Vj-Ago1a | XP_022710573.1 | Ago | NCBI |
|  | Vj-Ago1b | XP_022710581.1 | Ago | NCBI |
|  | Vj-Ago2a | XP_022688259.1 | Ago | NCBI |
|  | Vj-Ago2b | XP_022688515.1 | Ago | NCBI |
|  | Vj-Ago2c | XP_022711586.1 | Ago | NCBI |
|  | Vj-Ago2d | XP_022691691.1 | Ago | NCBI |
|  | Vj-Ago2e | XP_022691690.1 | Ago | NCBI |
|  | Vj-Ago3a | XP_022706928.1 | Piwi | NCBI |
|  | Vj-Ago3b | XP_022706929.1 | Piwi | NCBI |
|  |  |  |  |  |
| ***Ixodes scapularis* (Is)** | Is-Ago1 | XP_029823839.1 | Ago | NCBI |
|  | Is-Ago2a | XP_029837045.1 | Ago | NCBI |
|  | Is-Ago2b | XP_029839997.1 | Ago | NCBI |
|  | Is-Ago2c | XP_029837056.1 | Ago | NCBI |
|  | Is-Ago2d | XP_029849129.1 | Ago | NCBI |
|  | Is-Ago3a | XP_029836165.1 | Piwi | NCBI |
|  | Is-Ago3b | XP_029841517.1 | Piwi | NCBI |
|  | Is-Aub | XP_002399390.2 | Piwi | NCBI |
|  | Is-Aub/Piwia | XP_029847819.1 | Piwi | NCBI |
|  | Is-Aub/Piwib | XP_029847820.1 | Piwi | NCBI |
|  |  |  |  |  |
| ***Tropilaelaps mercedesae* (Tm)** | Tm-Ago1 | OQR70150.1 | Ago | NCBI |
|  | Tm-Ago2a | OQR76779.1 | Ago | NCBI |
|  | Tm-Ago2b | OQR66449.1 | Ago | NCBI |
|  | Tm-Ago2c | OQR66448.1 | Ago | NCBI |
|  | Tm-Ago2d | OQR70493.1 | Ago | NCBI |
|  | Tm-Ago3 | OQR71376.1 | Piwi | NCBI |
|  |  |  |  |  |
| ***Tetranychus urticae* (Tu)** | Tu-Ago1a | XP_015790045.1 | Ago | NCBI |
|  | Tu-Ago1b | XP_015790046.1 | Ago | NCBI |
|  | Tu-Ago1c | XP_015790047.1 | Ago | NCBI |
|  | Tu-Ago2a | XP_015785548.1 | Ago | NCBI |
|  | Tu-Ago2b | XP_025017682.1 | Ago | NCBI |
|  | Tu-Ago2c | XP_015785626.1 | Ago | NCBI |
|  | Tu-Ago2d | XP_015795466.1 | Ago | NCBI |
|  | Tu-Ago2e | XP_015795441.1 | Ago | NCBI |
|  | Tu-Ago2f | XP_025018514.1 | Ago | NCBI |
|  | Tu-Ago2g | XP_015781950.1 | Ago | NCBI |
|  | Tu-Ago3 | XP_015783287.1 | Piwi | NCBI |
|  | Tu-Auba | XP_015783303.1 | Piwi | NCBI |
|  | Tu-Aubb | XP_015783387.1 | Piwi | NCBI |
|  | Tu-Aubc | XP_015792164.1 | Piwi | NCBI |
|  | Tu-Aubd | XP_025016418.1 | Piwi | NCBI |
|  | Tu-Aub/Piwi | XP_015783386.1 | Piwi | NCBI |
|  |  |  |  |  |
| ***Dermatophagoides pteronyssinus* (Dp)** | Dp-Ago1 | XP_027205239.1 | Ago | NCBI |
|  | Dp-Ago2a | XP_027195170.1 | Ago | NCBI |
|  | Dp-Ago2b | XP_027205617.1 | Ago | NCBI |
|  | Dp-Ago2c | XP_027205616.1 | Ago | NCBI |
|  | Dp-Ago2d | XP_027201441.1 | Ago | NCBI |
|  | Dp-Ago2e | XP_027201245.1 | Ago | NCBI |
|  | Dp-Ago2f | XP_027205496.1 | Ago | NCBI |
|  | Dp-Ago2g | XP_027194229.1 | Ago | NCBI |
|  |  |  |  |  |
| ***Euroglyphus maynei* (Em)** | Em-Ago1 | OTF72339.1 | Ago | NCBI |
|  | Em-Ago2a | OTF75874.1 | Ago | NCBI |
|  |  |  |  |  |
| ***Sarcoptes scabiei* (Ss)** | Ss-Ago1 | KPM09009.1 | Ago | NCBI |
|  | Ss-Ago2a | KPM04840.1 | Ago | NCBI |
|  | Ss-Ago2a | KPM04151.1 | Ago | NCBI |
|  |  |  |  |  |
| ***Psoroptes ovis* (Po)** | Po-Ago1 | SZF06500.1* | Ago | NCBI |
|  | Po-Ago2 | SZF06480.1* | Ago | NCBI |
|  |  |  |  |  |
| ***Dermatophagoides farinae* (Df)** | Df-Ago1 | AUI38415.1* | Ago | NCBI |
|  | Df-Ago2a | AUI38416.1* | Ago | NCBI |
|  | Df-Ago2b | AUI38417.1* | Ago | NCBI |
|  | Df-Ago2c | AUI38418.1* | Ago | NCBI |
|  | Df-Ago2d | AUI38419.1* | Ago | NCBI |
|  | Df-Ago2e | AUI38420.1* | Ago | NCBI |
|  | Df-Ago2f | AUI38421.1* | Ago | NCBI |
|  | Df-Ago2g | AUI38422.1* | Ago | NCBI |
|  |  |  |  |  |
| ***Drosophila melanogaster* (Dm)** | Dm-Ago1 | NP_725341.1* | Ago | NCBI |
|  | Dm-Ago2 | ABB54719.1* | Ago | NCBI |
|  | Dm-Ago3 | ABO27430.1* | Piwi | NCBI |
|  | Dm-Aub | AGA18946.1* | Piwi | NCBI |
|  | Dm-Piwi | AAD08705.1* | Piwi | NCBI |
|  |  |  |  |  |
| ***Tribolium Castaneum* (Tc)** | Tc-Ago1 | EFA09197.2* | Ago | NCBI |
|  | Tc-Ago2 | EFA11590.1* | Ago | NCBI |
|  | Tc-Ago3 | EFA02921.1* | Piwi | NCBI |
|  | Tc-Aub | XP_008196303.1* | Piwi | NCBI |
|  | Tc-Piwi | EFA07425.1* | Piwi | NCBI |
|  |  |  |  |  |
| ***Caenorhabditis elegans* (Ce)** | Ce-Alg1 | F48F7.1* | Ago | WormBase |
|  | Ce-Alg2 | T07D3.7* | Ago | WormBase |
|  | Ce-Rde1 | K08H10.7* | Ago | WormBase |
|  | Ce-PPW1 | C18E3.7* | Wago | WormBase |
|  | Ce-PPW2 | Y110A7A.18* | Wago | WormBase |
|  | Ce-Sago1 | K12B6.1* | Wago | WormBase |
|  | Ce-Sago2 | F56A6.1* | Wago | WormBase |

*- The amino acid sequences of the query proteins are presented below and those of the Piwi domain (IPR02171/Pf003165) retrieved from the Pfam database appear in red within the sequence of the query proteins.

> Po-Ago1-SZF06500.1

MFPLGTQPSNNRGKAPLQPPTIVQHGQFAPGSLVVGSAGTGGNGGGGTVGPIGTVTPGSS
VVPSTSGLQGGGGNGGLGGSGNIAMGDIGYTGPRFISPRRPDYGRYGFPIQLRANHFQIT
MPRGFLHHYDVTITPEKCPRKINREIIEIMVQAYNKIFGNIKPVFDGRKNIYTRDDLPIG
RDKVELEVTLPGEGKDRVFRVAIKYVSKVNLDLLEDALRGNARTMPLDSIQALDVVMRHL
PSMTYTPVGRSFFTSPEGYFHPLGGGREVWFGFHQSVRPSQWKMSLNIDVSATAFYKAQP
VVDFLMEVLDFRDFDLIRKSLTDSQRTKFTKEIKGLKIEITHCGNMRRKYRVCNVTRRSA
QLQSFPLQLENGQTIECTVAKYFLDKYNMKLRYPNLPCLQVGQEHKHTYLPIEVCNIVPG
QRCIKKLTDMQTSTMIKATARSAPDREREINNLVQKADFNNDPYVQEFGLSISNMMMEVK
GRILPAPKIQYGGRTKQQAIPSYGVWDMRNKQFHIGVEIRTWAIACFTPQRTCREDSLRT
FTQSLQKISSDAGMPIIGQPCFCKYATGADQVEPMFRYLKQSFVGLQLVVCVLPGKTPVY
AEVKRVGDTVLGIATQCVQTKNVLKTSPQTLSNLCLKINVKLGGINNILVPNLRPKVFSE
PVIFLGADVTHPPAGDTKKPSIAAVVGSMDGHPSRYAATVRVQQHRQEVIRDLSTMVKEL
LVQFYRTTRFKPYRIILYRDGVSEGQFQQVLTHELIAIRKACVQLEEDYRPGITFIVTQK
RHHTRLFCVKEKEQVGKSGNIPAGTTVDVGITHPTEFDFYLCSHAGIQGTSRPSHYHVLW
DDNDFTADEIQLLTYQLCHTYVRCTRSVSIPAPAYYAHLVAFRARYHLVDLKEIDRLNRS
EVESNTSFPDSEPASTGEGTSHIPGTTFDDRTVDAMSRSIALHPNSFKVMYFA

> Po-Ago2- SZF06480.1

MDRNPKRGGGGRGGHGGGRGGYAGDRGGQGDSRGPQGSERGGRGRGGYGDDRGGQGDSRG
PQGSERGGRGRGGYGGDRGGQGDSRGPQGSERGGRGRGGYGGDRGGQGDSRGPQGSERGG
RGRGGYGGDRGGRQGGFDRRPEEPTNVPTGENYQIIRPSNMKKGDIGTEIELLSNFYKLE
INDKTLYQYDIDIKEKIDESKPVKDQNEKFLQKHSRNLLRNWIDLNKDIFDTVPYAYDGW
KILYSIEEFNLNEPFVKDELEYEIEGQKRKFSIIMKLVDKFSFQPLLDFYNQQTVNPAKL
LKILTAMEILLGNVCEISNQFYQRKYFDTNDFQINKYFQIAAGFAKAIRMTEFGLAVNLH
LKSACMISKTLNRVDELVVAYLGKQPDKLDDSNFCKLNKLCRHLKIFTNHCGKRTYVIDS
IVKYRPNSKTIDKEKGTIADYFLNTYNIRVKNYPLIKTTGKNSKELPMELCYLVDKQFLA
NSKIDERVQQNLLSASTHKPQVYFSKTTKYIERIKKDGHQLIDQFGLNLMIKPAKLIGRV
LPQPHQFRPGRSDKYHRTPDKTLKWVLFSMDPFFKSSNRGPNETLVKFADTIVREGSKVG
LKFDPSTNGCIFSVNKDLITQQDVISIMKNINKDFKDLDVAFIVIPSNNRNIYSSEVYNL
VKAFSERAVSNEESTSGYGFITQCLKSDNVKRLDTLLRKGYFQNLLNKVNGKVGGVNSIV
DTNEYRKNNMKFDPAKTMVIGIDVNHPSIDEKSSSSVAAAVGSYDDLFSKYTASLSVQPK
DRDEVITHLNIMIGELLQEYKKSNQRFPETLLVFRDGVSEGQFDKIKDIELPQIQKAALA
CNSKIKIVLFIVQKRHHTRFVSIRGGHGFKNDSHNVPSGTVVDNSIVDPKFDSFYVNSHF
SPLGTSKPTKYVIIRDDLKMSPNQLQQLCFFMCFNCVRFRGVIAIPTPIRYADLCAYRSK
LHIEAQTRVIKLNSKEKEEQLISKLNQWVKIDKKVQKLLYYC

> Df-Ago1-SZF06480.1

MFPMGTQPPNNRGKAPLPQMQPTTIGQHGQFTGSLVVGSAGTGGNGGGGTSGPIGSVAPG
SSVVPSTSGLQAGGGGNGSSGGSGSSAVGDPGYSGPRFISPRRPDYGHYGFPIHLRANHF
QITMPRGFLHHYDVTITPEKCPRKINREIIEIMVQAYNKIFGNIKPVFDGRKNIYTRDDL
PIGRDKIELEVTLPGEGKDRVFRVAIKYVSKVNLDLLEEALEGNARTMPQDSIQALDVVM
RHLPSMTYTPVGRSFFSSPDGYFHPLGGGREVWFGFHQSVRPSQWKMSLNIDVSATAFYK
AQPVVDFLMEVLDLRDYEQCRKNLSDSFRVKFTKEIKGLKIEITHCGTMRRKYRVCNVTR
RPAQLQSFPLQLENGQTIECTVAKYFLDKYKMKLRYPNLPCLQVGQEHKHTYLPLEVCNI
VPGQRCIKKLTDMQTSTMIKATARSAPDREREINNLVQKADFNNDPYVREFGLSISNMMM
EVKGRVLPAPKIQYGGRTKQQAIPAFGVWDMRNKQFHIGVEIRVWAIACFTPQRMCREDA
LRSFTQSLQKISNDAGMPIIGQPCFCKYATGADQVEPMFRYLKQTYVGLQLVVCVLPGKT
PVYAEVKRVGDTVLGIATQCVQTKNVTKTSPQTLSNLCLKINVKLGGINNILVPNIRPKV
FNEPVIFLGADVTHPPAGDTKKPSIAAVVGSMDGHPSRYAATVRVQQHRQDVIQDLSTMV
RELLLQFYRTTRFKPYRIILYRDGVSEGQFQQVLTHELIAIRKACVQLEEDYRPGITFIV
TQKRHHTRLFCAKEKEQIGRSGNIPAGTTVDVGITHPTEFDFYLCSHAGIQGTSRPSHYH
VLWDDNDFSADEIQLLTYQLCHTYVRCTRSVSIPAPAYYAHLVAFRARYHLVDLKEIDRL
SQAEIESNTSTSFSENEPHNPTGEGTSHHPGTNFDERTTAAMSRSIALHPNSFKVMYFA

> Df-Ago2a-SZF06480.1

MVDNGINNGCGSSATSGSGSGGQAGRSMLIQTNHFMITYDPDKIIYSYYVKINPIISNNN
DDHHHHSQQRLKNMAKTIQQQQTTPNRSHNNGGGNELLSRKIIQRLIEMNNGDGNIFHNV
LCVYDGASSMLTNFRLPIIDDKLIKFRVTLPANDYYHNEQEFEVCIKLIKTVAYSTIEDF
YRNPMNDGYVEVVTAVNLIIRHLLLKKRFLIGRSNFHHHSSVDNRINLSSLKELSFGISS
SVQTCSAGLQLIMDRCCTPFIKPFMIDDCIRNFLQEMSSNTTGSNNVNHHHNHHHHHHHH
QQQQQRWNDFYRKRLEPILKDYYFEVTNLQRSRRYRIGGITTESAKKVMFTPNNDNSEQQ
QQQRKMISVADYFHQQYGQLQRPDLPCTKVRRGKNEFIYFPVEICQIIADQKARKLTIKE
KADLIRKAASINPIDRLDEVRNSVEELINIDNNENYLKEFGFNISTQPITIRARVLDSPK
LLEGDDRPIKLTNGKWRYRKFFQPIALKRWILVKLMHFDDSTVEKFLDTFIRNGEQLGMQ
IDMPIRMDYDFDIATLANFLQKLQHTYSSGSGSEQQLQLLFFIGCNTEWQHSAIKKCGDV
DFGLVTQCIRQTNVQRINQSIAVNILHKINAKLGGINVTLDYGKLLQADDYRKTMAIGVD
VAHPSPSEKNLPSIAAMVANVDESFARYTSSVKIQKFFRQEIIQELDKMLIDLLRAYEME
TNQLPERIILYRDGVSEGQFQVVFNEEILLIKETLARYRSGHKFRLTFVIVQKRHHSRFI
PDNSKDGVGRFYNIPPGTVVDTDVVHPRRFDFYLCSHAGIQGTSRPSHYCLLVDENEMSA
DHIQNFTNVLCHLYARCSRSISIPTPVFYAHLAAFRAREHIQAATSLMMLSMTNNRNQNF
NNQQQQQQQQQQQQQQDAAKMLNYFSTTTTTTSATAQSFLSSSSSMMTTPNNNNNNKNNN
NNDDDDILSSSPRIHYTKHRVPCLDNFDSFVEMSPTMRSSMYFC

> Df-Ago2b-AUI38417.1

MERKHSLSSISSRGSRFPDSKRWRNQEERRDYNDNYGRTSGHRSSRSFRDSGHDDDHRHS
INRSRRDYHDDSRGFHHDEKRQDRSIRNVTDHERRRYGSSCSSPRGRRPYSRGGDNKPSH
HSSLPNFPISEEYAIRIANNPIVNHGKEQKLTGNHFKLSTEKIMVYHYDVQISQRVKNES
LIPKNEPPTTAGGEDDSHNTGPTPIVNERQMVFLKRNSHRIVEQFVEKNSKFFTTHPYVY
DGWANFYLCAALRTEDLATDFEFIAGDGGGGSGNRQRPSMNERITSVRLKLVDKIDLGIV
EEYFQRRCRHLPNQIINICNLVFHNILRDNFVHNHSRYFNMGNVSEDESLPYLEFVFGFE
LSVRMAQIGLSLVVNPRVGCLFSRKHDKLIDIIRAMQPGFPRQIDFNGINAILKHITIYT
EHGDRKRYYNFHSLIPEKPSNLRFKDASGEELSVFEYFDQRYQVNLNCEYPLVLAKSKGG
VSRSQKNHLPLELCFIGKQQFINPTKTSREIQTVALRKSELKPEKFFHCIQTYVDKIIGT
DSRLLREFGIHIHNRPACFKGYQLEQPTFINPNQFRITESGSNHSWALISFDPNISHRTL
DQFSYMLNKEAGKMNIQMGRLIRTLTYDIRSIGQIRPAFAEINRAGIKFCLAVIPDRNSA
LDPTQIYGACSSVCTQEFSITLQCLNASRVRNPPRGYFNNLLFEINGKRGGQNTVIEPEF
FRSQLPTIDFSKTMIIGLDVNHPGFRESVPISIAAAAGTYDSLLTQYISTARVQKFDRTE
MIHHLDEIIKELLAHYRFQNERRVPEHLIIFRDGVSEGQFQTVRNEEIPLIRKAMEEKGS
KNMTLTLIIVQKRHNTRFVTTEPYEKDGRSRQMTRNVPSGTVVDNTIVEPNFDIFYVNSH
FSNLGTSRPTKYIVSVNELNLSNAELQRLCFFVCFNCVRHKMPLSIPTQVMYADLCAYKS
KIHIMHQLLTDRSFRDQRSEDFDFDDDRPSLESIEVENRLIQRYQQFVHIPNNSKDCLFY
V

> Df-Ago2c-AUI38418.1

MAPQNFTSYPESEAYDIVLPNEIEHGVKGRKIELLANYMRISIKPTMVYHYNIDFNFKDD
EERVAINANNERKQKYFANNALELVTRFVEINEEKMNQLNEFVYDIGKNIYTTKEIDLNE
LTKNFEANIGGQRKRFVAKIVNVQKIDLTQIVDFYEGRTSEAPQVIIHFLELLIQNVSLS
KFHPHRRNLFDIESGVIRSPKNFVKFVQGFSTAVHLTEMGPSLNLHLKTSCMISLEADTL
LQLVAMISEGRDPQHLNPNEIENISKIIRGLAVNTDYTGRKQKYIVKAFAARRPHEVTFM
MKKNGTDEETPISVHDYFLTKYRISVKDYPVVQMCGKRDTVIPLELIYLVEKQFLNNSKI
DSIIQNELLRTATHKPLIYFNHLSRFTQEIVKSNPERMAQFGTDLSPKPVRFCGRVLDEP
RVRGGNRNERFASASVARWAFFSFDEKFGERDVDSIVLELKQTANRFGMNLSNCIEKQVV
PIDNRSLDVVKNVFANILKKLPNLNLLFVALPHIPFLYNAVKHFGDQKFGIVTQCLSVMK
AKRRNRGYIENLLLKVNGKLNGQNSFIERQDLQSLSIDHTKTMAIGVDVNHPSYTEKVQS
SIACAIGSYDHEFTRYSASVRVQKREKEEMIYVLEEMVDELLDEYEKRNKYLPENFVIFR
DGVSDGQFKYAENEINQIRTAIRRRTKTGKLVYIVTQKGHQTRFVLQNPSGSADRPVYNV
PPGTVVDHTITDPTKCMFFMNSHFSSLGTSRPMKYVVLHNDYERRKMNMDDLEKLCFYLC
HNCTRFRGPIAMPVPVRYADLCAYRSKLHLEAQHASKNIPAESQEEFERHVISQLNKLVK
LNDKIKNSLFYC

> Df-Ago2d- AUI38419.1

MSHRDFGGGRGRGGGRGGGRGFHGGQGDRGPQRGGGGGGRGYGGQGDRGPQRGGGGGGRG
FGGQGDRGGRGGQGGPYRGRGGGSSGHGHGPPVSIPDGEQYQLIRKSQMAKSETGVPIEL
LSNFYRMIVSDKIVYHYDIDIKEKADEKKPAREQNEKFLKKYARSLLPEWTKKNGQIFEK
INYIYDGWKNLYTVQELHLNQGFIEELTYEIDARKRAFEIKIKLVDKISLRPLMDYYAKK
TSKVEPKIFSVLDVLFNNISEISFTYYQRKFFDTNSVRDCHNTNWCQFAPGFANSIRMTE
FGPAMNIHLKTACLISKTLNRVDEMVCAFKRKQPNELSDYELRDLSKFCRHMKIFTNHCG
KRTFLIERFEKITPQQKKIDDNKGTIADYFRNTYGINVKSYPLIKTTGKIAKYLPMELCY
LVDKQFLANSKIDDRIQRELLEISTHRPDVYFTKATTYASKIKQDGTGFLKDFGLDLMMK
PAKLIGRVLPEPKQFHSRRDDKYYVTPRKELKWVLFSLEPSIRKNDLQSLADQIIGTGEK
VGLRFMPKSNNILATEEIRNHKDVIGIMKNINKEYNKANSPLDVAFIVIPSYNRNVPSVE
IYNLVKAFSERAVSDSNNGGFGFITQCMKSDNIRRPKGGYFQNLLLKVNGKVGGVNSIVD
PNEFKLKQMKFDPSSTMVIGIDVNHPSVTETSNSSVAAAVGSFDSLFSKYTASLTVQPKD
RDEIITRLNIMINELLQEYKQKNGKFPETLLIFRDGVSEGQFDKIKDKELPLIQKAVNDC
GGKMKIVLFIVQKRHHTRFMSIKGGHGPRHDSYNVPSGTVVDNSIVDPKFDSFYVNSHFS
PLGTSKPTKYVIIRDDLKLQPNQLQQLCFFMCYNCVRFRGVIAIPTPIRYADLCAYRSKL
HIEGQCKLSMINPKDAEQAIITQLNQWVKIHKKVQNFLYYC

> Df-Ago2e-AUI38420.1

MSFRGRSRPRFIFRPAALPVTSSSSSSSLSTTTTTTTTTSPLSPLSSLSPSLPIDTPTRP
TTEVVEEQTREQLRRMQIQAPSFRDSGDNIPVTRAEHLERKSPPQIKPQSKGGTTVIRRD
FSNYPEAQSYPIVYSGKFESGTNGRKIELLANFMLINVHPTIVFQYDVDFKFKNSERQKT
ISADENERIQRFFAKHAPKLVGRFVNLNQNVFTGIKHIYDNGKNIYATEDIDSSILVKNI
DAEIDGRKKEFIVEFSKVQIIDLSTIIGFYSGKISNIPEMAISFLEVLFQDICLNKFQLH
RRNLYDVENGQTSGPNSWLRFVQGFTTAVHRTQIGPSLNLHLKTSCLISLSTESLVQFVE
MISDCRNIRNLSSNDLDRVNKIIRGLEVYSTHMGRKMTYKIKALVSKKPREVTFMFRERN
NGNGDDNNNDHERLISVYDYFMMKYQMELEDYPTVQMMGRSNFFMPLEILHMVDKQFLNQ
SKIDPLIQNDLLHASTHKPLVYFNHLSRFATEIAQLDTDKMNHFGANFSPKPVRLNGRVL
DPPHLIGGGRNDRFASTVGSKQWAFFSFDEKFDKRDVANIVSELKQTGHRFGLDLSKCVE
TQVVPIDNRSLNVVKNVFANILKLLPDVNLLFILLPQIPYLYNIVKHFGDQKFGIITQCL
SSSKAKRRSRGYIESVLLKVNGKLNGQNSYIDTRDLKSLPFDHMKTMAIGIDVNHPGSGE
KVLNSIACAIGSYDQHFTRYSASIRVQKREKEELIHVLEEMINELLDEYEKINKYLPENF
IVFRDGIGEGQFKSAQDEIDQIRRAVRLRVKNYKLLFIVTQKRHQTRFVLSKPSGPSDRP
VHNVPSGTVVDTTIIDPNYCMFYINSHFSPLGTSRPLKYVVLHNDYERRQMSMDDLQKLC
FYLCHNCTRFRGGPIAMPIPVRYADLCAYRSKLHLEGQHESRHIPAENQEEFERHIITRL
NELVKLNDKMKNTLFYC

> Df-Ago2f-AUI38421.1

MSQNRGKPKRSGFASDGGGGGDRFAGNNNNRYPRNNDNRQRSFDASPVEFPESQQYPIRR
SLSPKVDQKNSLNLVGNYYKLDITEKEIYHYNVQISQPPSINQTQKNDSRSEQNREKFLR
NNSREILKEFLSKNPTLFQDIPFIYNGWENLYTTKKIQLDNDYVNEFEYRVGNGKPDRSV
PKTNVEIVFKEKINLKIIEDFYNGKVNEIPNKIIDILNKLFAKILMEYYTVRGQCYFDMN
QPRSVRELSFIEFVYGFELSTRMAQIGICLVIQPRVECLISSKCKTLMDLINEYDYKSGE
NITNFEKINFKKINSFIKNLSIYTEHGNQKRSYKIKELIAIRLNEMKLGDDDDNMTILDY
FNKNYPEVKINPKLPMVQMTKPHVYMPLDLCYIDEKQFVDNTKNYPNIHKVFMKECALKP
ESFFNKIQSYVDHIGKIGSTILGEFGVNLQNKLVKFNGYQLDKPRMVVRNPRAITKSASI
VPWAFISFDEKISNGIIDDFIDGLLNEAEKMNSNLPMGLKYSDFKRIKNLNDIENVLKKL
KTECKIEFCFVVVPEHHNYLTPSDIYNAASSIGCKDLGLKIQCLNGFRVANVPGGYFNNL
LFEVNGNLGGQNTVVEPRYFQQQIGAKIQLNETMIIGIDVNHPGFFECSQNFRVSIAAAV
GTTNVEMTDFGNCFRIQKRERTMEIIEQLAEMIKELLEKYKMTNGKYPKNLIIFRDGVSE
SQFDQVKMIELQDIRRLIEQTKLKIRFTLIIVQKRHHTRFVKTMPTSSDANQKGPPTRNV
PCGTVVDNSICEPNFTVAYVNSHFSRLGTSKPTKYIVIENQLQFTNSELHKLAYLVCYNS
VRFSSPLSIPTPIKYADLCAYKCKIHLLHELKKNPIQSTDEWNVEHLQQMITFHDPKAQD
SFFYL

> Df-Ago2g-AUI38422.1

MVQQIDLTHIDEYYKMTASDLPEVMRHFMELLFQYCYTCRFFSHQHGFYDTDSSSLMRGP
MEWVKFASGFSHGVCLTEMGLSLNIHLKTSCLISPSVDELSKLVTLIAGGRDPSQFGPGD
IVAANKIIRGLKIYTTHTGRKTCYTIKGLTVQKPNEHKFTKKTKDQDEGQIVSIQEYFLE
KYKVRVQNYPLVSTVGKEQFIPLELCRMVDKQFLNNSKIQADANIPNELLRVSTHKPLIY
FDKLSKISNQIPSLSPELMSEFKTDFFTKPIRFTGRVLDTPRQMNAQRMEPFFRSIPKTP
WAFVSLDINFNKPDVENIVSELVTTAKRYKLDLSNCRNKIVTPVNPGNLAQVDLIFAKIK
TEIPDLKLLFVALPQCAGMYNMVKFCGDQKYGFATQCLNSMKAKQRGRGYLDNVLLKVNG
KIGGQNSIIDASEWKKLPFDHAKTMVCGIDVNHPGQTERIESSIAAVVGSYDDLFTLYTA
SICSQTKRCDEEITCLEPMITELLDAYHQRNKFYPQTLAVFRDGVSDGQFQYAEKEIKQI
RTAFRKKVPKGKIIFIVVQKGHRTRFVLSKPSGPNDRPVYNVPSGTVVDHTIVDPSQHMF
FLNSHFSQLGTSRPMKYVILENDFDKKIFNNDALQKFVFYLCHNCTRFRGGAIALPTPVR
YADLCAYRAKIHVEAQIEKLCMPRGQIIQGDYEKKLIDRLNELVKIHQSLKRVLYYA

> Dm-Ago1-NP_725341.1

MSTERELAPGGPAQLHPHTLPLTFPDLQMTSTVGIIGKVYESQWTPSPTRPQSPSQAQTS
FDTLTSPPAPGSSVNPTAVTSPSAQNVAAGGATVAGAAATAAQVASALGATTGSVTPAIA
TATPATQPDMPVFTCPRRPNLGREGRPIVLRANHFQVTMPRGYVHHYDINIQPDKCPRKV
NREIIETMVHAYSKIFGVLKPVFDGRNNLYTRDPLPIGNERLELEVTLPGEGKDRIFRVT
IKWQAQVSLFNLEEALEGRTRQIPYDAILALDVVMRHLPSMTYTPVGRSFFSSPEGYYHP
LGGGREVWFGFHQSVRPSQWKMMLNIDVSATAFYKAQPVIDFMCEVLDIRDINEQRKPLT
DSQRVKFTKEIKGLKIEITHCGQMRRKYRVCNVTRRPAQMQSFPLQLENGQTVECTVAKY
FLDKYRMKLRYPHLPCLQVGQEHKHTYLPLEVCNIVAGQRCIKKLTDMQTSTMIKATARS
APDREREINNLVKRADFNNDSYVQEFGLTISNSMMEVRGRVLPPPKLQYGGRVSTGLTGQ
QLFPPQNKVSLASPNQGVWDMRGKQFFTGVEIRIWAIACFAPQRTVREDALRNFTQQLQK
ISNDAGMPIIGQPCFCKYATGPDQVEPMFRYLKITFPGLQLVVVVLPGKTPVYAEVKRVG
DTVLGMATQCVQAKNVNKTSPQTLSNLCLKINVKLGGINSILVPSIRPKVFNEPVIFLGA
DVTHPPAGDNKKPSIAAVVGSMDAHPSRYAATVRVQQHRQEIIQELSSMVRELLIMFYKS
TGGYKPHRIILYRDGVSEGQFPHVLQHELTAIREACIKLEPEYRPGITFIVVQKRHHTRL
FCAEKKEQSGKSGNIPAGTTVDVGITHPTEFDFYLCSHQGIQGTSRPSHYHVLWDDNHFD
SDELQCLTYQLCHTYVRCTRSVSIPAPAYYAHLVAFRARYHLVEKEHDSGEGSHQSGCSE
DRTPGAMARAITVHADTKKVMYFA

> Dm-Ago2-ABB54719.1

QSQGQYQSRGPPQQQQAAPLPLPPQPAGSIKRGTIGKPGQVGINYLDLDLSKMPSVAYHY
DVKIMPERPKKFYRQAFEQFRVDQLGGAVLAYDGKASCYSVDKLPLNSQNPEVTVTDRNG
RTLRYTIEIKETGDSTIDLKSLTTYMNDRIFDKPMRAMQCVEVVLASPCHNKAIRVGRSF
FKMSDPNNRHELDDGYEALVGLYQAFMLGDRPFLNVDISHKSFPISMPMIEYLERFSLKA
KINNTTNLDYSRRFLEPFLRGINVVYTPPQSFQSAPRVYRVNGLSRAPASSETFEHDGKK
VTIASYFHSRNYPLKFPQLHCLNVGSSIKSILLPIELCSIEEGQALNRKDGATQVANMIK
YAATSTNVRKRKIMNLLQYFQHNLDPTISRFGIRIANDFIVVSTRVLSPPQVEYHSKRFT
MVKNGSWRMDGMKFLEPKPKAHKCAVLYCDPRSGRKMNYAQLNDFGNLIISQGKAVNITL
DSDVTYRPFTEDERSLDTIFADLKRSQHDLAIVIIPQFRISYDTIKQKAELQHGILTQCI
KQFTVERKCNNQTIGNILLKINSKLNGINHKIKDDPRLPMMKNTMYIGADVTHPSPDQRE
IPSVVGVAASHDPYGASYNMQYRLQRGALEEIEDMFSITLEHLRVYKEYRNAYPDHIIYY
RDGVSDGQFPKIKNEELRCIKQACDKVGCKPKICCVIVVKRHHTRFFPSGDVTTSNKFNN
VDPGTVVDRTIVHPNEMQFFMVSHQAIQGTAKPTRYNVIENTGNLDIDLLQQLTYNLCHM
FPRCNRSVSYPAPAYLAHLVAARGRVYLTGTNRFLDLKKEYAKRTIVPEFMKKNPMYFV

> Dm-Ago3-ABO27430.1

MSGRGNLLSLFNKNAGNMGKSISSKDHEIDSGLDFNNSESSRERLLSSHNIETDLITTLQ
HVNISVGRGRARLIDTLKTDDHTSNQFITSESKENITKKTKGPESEAIASENGLFFPDLI
YGSKGSSVNIYCNYLKLTTDESKGVFNYEVRFFPPIDSVHLRIKYLNDHKDKLGGTKTFD
GNTLYLPILLPNKMTVFISKAEDVELQIRILYKKKEEMRNCTQLYNILFDRVMKVLNYVK
FDRKQFDPSRPKIIPLAKLEVWPGYVTAVDEYKGGLMLCCDVSHRILCQKTVLEMLVDLY
QQNVEHYQESARKMLVGNIVLTRYNNRTYKINDICFDQNPTCQFEIKTGCTSYVEYYKQY
HNINIKDVNQPLIYSIKKSRGIPAERENLQFCLIPELCYLTGLRDEVRSDNKLMREIATF
TRVSPNQRQMALNKFYENVSNTPAAQEILNSWGLSLTNNSNKISGRQMDIEQIYFSKISV
SAGRSAEFSKHAVTNEMLKVVHLSKWIIIHLRNYRQAATSLLDNMKQACESLGMNISNPT
MISLDHDRIDAYIQALRRNITMNTQMVVCICHNRRDDRYAAIKKICCSEIPIPSQVINAK
TLQNDLKIRSVVQKIVLQMNCKLGGSLWTVKIPFKNVMICGIDSYHDPSNRGNSVAAFVA
SINSSYSQWYSKAVVQTKREEIVNGLSASFEIALKMYRKRNGKLPTNIIIYRDGIGDGQL
YTCLNYEIPQFEMVCGNRIKISYIVVQKRINTRIFSGSGIHLENPLPGTVVDQHITKSNM
YDFFLVSQLVRQGTVTPTHYVVLRDDCNYGPDIIQKLSYKLCFLYYNWAGTVRIPACCMY
AHKLAYLIGQSIQRDVAEALSEKLFYL

> Dm-Aub-AGA18946.1

MNLPPNPVIARGRGRGRKPNNVEANRGFAPSLGQKSDPSHSEGNQASGGNGGGGDAQVGP
SIEKSSLSAVQMHKSXGDPRGSVRGRRLITDLVYSRPPGMTSKKGVVGTHITVQANYFKV
LKRPNWTIYQYRVDFTPDVEATRLRRSFLYEHKGILGGYIFDGTNMFCINQFKAVQDSPY
VLELVTKSRAGENIEIKIKAVGSVQSTDAEQFQVLNLILRRAMEGLDLKLVSRYYYDPQA
KINLENFRMQLWPGYQTSIRQHENDILLCSEICHKVMRTETLYNILSDAIRDSDDYQSTF
KRAVMGMVILTDYNNKTYRIDDVDFQSTPLCKFKTNDGEISYVDYYKKRYNIIIRDLKQP
LVMSRPTDKNIRGGNDQAIMIIPELARATGMTDAMRADFRTLRAMSEHTRLNPDRRIERL
RMFNKRLKSCKQSVETLKSWNIELDSALVEIPARVLPPEKILFGNQKIFVCDARADWTNE
FRTCSMFKNVHINRWYVITPSRNLRETQEFVQMCIRTASSMKMNICNPIYEEIPDDRNGT
YSQAIDNAAANDPQIVMVVMRSPNEEKYSCIKKRTCVDRPVPSQVVTLKVIAPRQQKPTG
LMSIATKVVIQMNAKLMGAPWQVVIPLHGLMTVGFDVCHSPKNKNKSYGAFVATMDQKES
FRYFSTVNEHIKGQELSEQMSVNMSCALRSYQEQHRSLPERILFFRDGVGDGQLYQVVNS
EVNTLKDRLDEIYKSAGKQEGCRMTFIIVSKRINTRYFTGHRNPVPGTVVDDVITLPERY
DFFLVSQAVRIGTVSPTSYNVISDNMGLNADKLQMLSYKMTHMYYNYSGTIRVPAVCHYA
HKLAFLVAESINRAPSAGLQNQLYFL

> Dm-Piwi-AAD08705.1

MADDQGRGRRRPLNEDDSSTSRGSGDGPRVKVFRGSSSGDPRADPRIEASRERRALEEAP
RREGGPTERKPWGDQYDYLNTRPAELVSKKGTDGVPVMLQTNFFRLKTKPEWRIVHYHVE
FEPSIENPRVRMGVLSNHANLLGSGYLFDGLQLFTTRKFEQEITVLSGKSKLDIEYKISI
KFVGFISCAEPRFLQVLNLILRRSMKGLNLELVGRNLFDPRAKIEIREFKMELWPGYETS
IRQHEKDILLGTEITHKVMRTETIYDIMRRCSHNPARHQDEVRVNVLDLIVLTDYNNRTY
RINDVDFGQTPKSTFSCKGRDISFVEYYLTKYNIRIRDHNQPLLISKNRDKALKTNASEL
VVLIPELCRVTGLNAEMRSNFQLMRAMSSYTRMNPKQRTDRLRAFNHRLQNTPESVKVLR
DWNMELDKNVTEVQGRIIGQQNIVFHNGKVPAGENADWQRHFRDQRMLTTPSDGLDRWAV
IAPQRNSHELRTLLDSLYRAASGMGLRIRSPQEFIIYDDRTGTYVRAMDDCVRSDPKLIL
CLVPNDNAERYSSIKKRGYVDRAVPTQVVTLKTTKKPYSLMSIATKIAIQLNCKLGYTPW
MIELPLSGLMTIGFDIAKSTRDRKRAYGALIASMDLQQNSTYFSTVTECSAFDVLANTLW
PMIAKALRQYQHEHRKLPSRIVFYRDGVSSGSLKQLFEFEVKDIIEKLKTEYARVQLSPP
QLAYIVVTRSMNTRFFLNGQNPPPGTIVDDVITLPERYDFYLVSQQVRQGTVSPTSYNVL
YSSMGLSPEKMQKLTYKMCHLYYNWSGTTRVPAVCQYAKKLATLVGTNLHSIPQNALEKK
FYYL

> Tc-Ago1-EFA09197.2

MYPGPGGQSWQTSPPHQSASPPPQTAFADPLSATAPGTASTAVAVVGATSTALATVPPTT
DPPVFQCPRRPNLGREGRPIGLKANHFQVTMPRGFVHHYDVSIQPDKCPRKVNREIIETM
VHAYGKIFGNLKPVFDGRNNLYTRDPLPIGNSREELEVTLPGEGKDRLFRVTIKWVAQVS
LYGLEEALEGRTRQIPYEAILALDVVMRHLPSMSYTPVGRSFFSSPEGYYHPLGGGREVW
FGFHQSVRPSQWKMMLNIDVSATAFYKAQPVIEFMCEVLDIRDINEQRKPLTDSQRVKFT
KEIKGLKIEITHCGTMRRKYRVCNVTRRPAQMQSFPLQLDNGQTVECTVAKYFLDKYKMK
LRYPHLPCLQVGQEHKHTYLPLEVCNIVAGQRCIKKLTDMQTSTMIKATARSAPDREREI
NNLVRRADFNNDPYVQEFGLTISNNMMEVRGRVLPPPKLQYGGRVASLSGQVGWHSKQQA
MPNQGVWDMRGKQFFTGVEIRVWAIACFAPQRTVREDALRNFTQQLQKISNDAGMPIIGQ
PCFCKYATGPDQVEPMFRYLKSTFQSLQLVVVVLPGKTPVYAEVKRVGDTVLGMATQCVQ
AKNVNKTSPQTLSNLCLKINVKLGGINSILVPSIRPKIFNEPVIFLGADVTHPPAGDNKK
PSIAAVVGSMDAHPSRYAATVRVQQHRQEIIQELSSMVRELLIMFYKSTGGYKPHRIILY
RDGVSEGQFLQLLQHELTAIREACIKLESDYKPGITFIVVQKRHHTRLFCADKKEQSGKS
GNIPAGTTVDVGITHPTEFDFYLCSHQGIQGTSRPSHYHVLWDDSHLDSDELQCLTYQLC
HTYVRCTRSVSIPAPAYYAHLVAFRARYHLVEKEHDSGEGSHQSGSSEDRTPGAMARAIT
VHADTKKVMYFA

> Tc-Ago2-EFA11590.1

MAPLPDGPDPSTKQKQPPTFAPQKESSFSTQKLKSSLSDCLSQTVIIKPGVKGRPIKIES
NHLSLNVGTLTEAYHYDVSITPDTPKSFLRDVMNLFARKHYPKNHPAFDGRKNLYSPKKL
PLPNDTMSDTIELEGENKKRGFKVVVKLARTVDLSPLRDILQTRQSPQDALQCLDIVLRN
APSNSCISSGRCFFTPPREGQILRLGDGMEMYYGFYQSAIRGWKQPLLNVDVVHKAFPEA
LNVLDLVCELGSDYRNTMTRQDLNQPLTDFVQKALEKFLKQLKVTYEIPGQSGSRRIFRV
NGLRAPPSQARFTLGDGKVTTVEKYYQEVKRCRLQYPHLPTLWVGSRQREVLIPLEFCTV
VSGQVVNRKMNENQTSVMIKKAATSTDVRKDKIMQVLRKANYNSDPCVREFGFSVNNSFE
KLDGRVLQPPTLLYARKAEVTPSKGVWRADMNRFFVGAIVHKWTIVSCTRHPERGEQLAD
MIFRMASSNGMQITSKATGPFQHLGGRQNLRDIIDYFKRKQDHDLIIVVVPNSGPQYSLV
KQAAELNVGCLTQCIKERTIAKLNPQIIANILLKINSKLNGTNHILSSRLPIMSRPCIIM
GADVTHPGPDAKDVPSVAAVTASHDPNAFQYNICWRLQPPKVEIIEDLCAITVEQLMFFY
RKTRHKPETIVFFRDGVSEGQFAEVRRAEISAIHQACKKLQREGYEPRITFLVVQKRHHT
RLFPTNPRDSEDRNNNVPAGTCVDTHITNPMMQDFYLVSHASIQGVAKPTKYCTLWDDNN
MSNDDIEELTYYLCHMFTRCNRSVSYPAPTYYAHLAAARAKVYVENVKLDLTQLKTHQQK
CQIQESIVKEKPMFFV

> Tc-Ago3-EFA02921.1

MEQKPAPKGRGALLEMLKKHKEARAGGAGEPVEEQAPPKTRGRAMLLQKIQEAKERKAGG
DSGQLSTPGPSTVPSETRRGVSGVTKALGEVAITASETCSYRGESGTPIKATANYILLNV
EKDRGVFEYEVRFQPDIDAKSNRIKLVNQALGELSTTKVYDGDVCLYLPCLAFSPRQEFE
SVIPNTETPVTTTLIYKRKRKLSECLHLYNVLFKRIMHILLYQRMGRNYFSPDHKYLVPQ
HKLEVLPGFCVHVDEMEGGLMVCLDTQHRVIRSQTVYELFHEIRATNPRNFREEVTKNVI
GACVLTKYNNRTYIIDDIAWNMNPKDTFEDRSKGPSCFIDYYREHYNIRIEDVDQPLLIT
RQVKQSPDGKIERMICLIPELCYLTGLTDAMRNDFKVMKDVAAFTRITPNQRMLALRTYL
DRVRQSEKAKQVLSGWGLSLADDTVDVKARVLPQEAIYFGGPDAEAHKYTGGTDWNKAIS
DNKLTGPVNITNWQLYYTRRDQKYAANFAQTIVRLGKGMGCVIQDPRHIVLDDDRTETYM
TAIRDNVANTQVAVFICPTLRADRYSIIKKMCCVNIPVASQVILSKTLSNPQKVRTIIHK
IAMQITCKLGGTLWSVKIPVSGWMVCGIDVYHGANNQSVCGFVASINGSMTKYFSKAMFQ
DGEIGDYFKMPFRQMLQAAKDREGAFPSKVIVFRDGVGDGQLEHCRKYEITQLQEVIKEL
NIETTITFVVVQKRINTRIFRTVNETNFENPPSGTVVDNMVTRRQFYDFFLVPQSVRQGT
VNPTHYVVLVDEGNIKPDHLQRLAYKLCHLYYNWSGTIRVPAPCLYAHKLAAIVGQYIKK
TPSTQLDDKLFYL

> Tc-Aub-XP_008196303.1

MEARGKGRGYGRARGQAAGQQQQQQQRPGGRPGGDQGQPAPRGLPQSAWVRPAQPAGSAW
PRPQMGSAPQQQKPQVAGRGERQEFDQGAVPRRVIAGEGDQGNQEGSQGAARGGGASSVR
GRVVRKEILYTRPQNLKSKKGTIGTPINLIANYLPLIKQGKWCLYQYRVDMAPDVDNTNK
RKELVRVAVKDLLKGGYLFDGTVLYTTQRINNDSVDLFVDNSGENVRITIRLVGDLAWGD
MHYIQLFNIIIRKCLKLMGLQQVGRNYFMPDNKIVISEHKIQLWPGYFTSMRQHEKDILL
NVDLQFKFMRTDTVYDNLLECQGANARKEFQSKIIGSVVLTHYNNKTYKIDDVDFNSTPA
HTFKLKDGSETTFKDYFKKKYNVDIRVKDQPMLISRSKPREIRVGVPETVYLVPELCLMT
GLTDRQRENFNLMKMLATHTRIGVEGRIKKLMEFSQKLHNKPDVVNEIRRWGLDVGNSLV
RFQGRVLPQETVVGGNDAKYSAGPQADWTKELRSRPMLYMPKMERLAVVCSHRNKSATQD
FIQLLAKTAGGMRWSLGNPKIFDIQDDRSGSYIEQIEKIINMNQPTMILVILPNNSTERY
SAIKKKCYVDRGIPTQMFVARNLTSKGVMSIATKVAIQMNCKIGGAPWCVPIPLSGLMVV
GYDVCRDTVNKKKSFAGIVGSLDKNISRFYNICCEHKMEEELSDNFAAAVVLLCKQYKEQ
NGHYPERILIYRDGVGEGQLPFVVEHEVANIKRKLQEEIYINGEVKMAFVVVSKRINTRI
FTEKDNPPPGTVVDDVITLPERYDFYIVSQCVRQGTVAPTSYNVIEDSMGLPPEKLQYLT
YKLTHMYYNWSGTVRVPAPCQYAHKLAFMVSQYIHRPAHHDLDNVLYYL

> Tc-Piwi-EFA07425.1

MEARGKGRGYGRARGQAAGQQQQQQQRPGGRPGGDQGQPAPRGLPQSAWVRPAQPAGSAW
PRPQMGSAPQQQKPQVAGRGERQEFDQGAVPRRVIAGEGDQGNQEGSQGAARGGGASSVR
GRVVRKEILYTRPQNLKSKKGTIGTPINLIANYLPLIKQGKWCLYQYRVDMAPDVDNTNK
RKELVRVAVKDLLKGGYLFDGTVLYTTQRINNDSVDLFVDNSGENVRITIRLVGDLAWGD
MHYIQLFNIIIRKCLKLMGLQQVGRNYFMPDNKIVISEHKIQLWPGYFTSMRQHEKDILL
NVDLQFKFMRTDTVYDNLLECQGANARKEFQSKIIGSVVLTHYNNKTYKIDDVDFNSTPA
HTFKLKDGSETTFKDYFKKKYNVDIRVKDQPMLISRSKPREIRVGVPETVYLVPELCLMT
GLTDRQRENFNLMKMLATHTRIGVEGRIKKLMEFSQKLHNKPDVVNEIRRWGLDVGNSLV
RFQGRVLPQETVVGGNDAKYSAGPQADWTKELRSRPMLYMPKMERLAVVCSHRNKSATQD
FIQLLAKTAGGMRWSLGNPKIFDIQDDRSGSYIEQIEKIINMNQPTMILVILPNNSTERY
SAIKKKCYVDRGIPTQMFVARNLTSKGVMSIATKVAIQMNCKIGGAPWCVPIPLSGLMVV
GYDVCRDTVNKKKSFAGIVGSLDKNISRFYNICCEHKMEEELSDNFAAAVVLLCKQYKEQ
NGHYPERILIYRDGVGEGQLPFVVEHEVANIKRKLQEEIYINGEVKMAFVVVSKRINTRI
FTEKDNPPPGTVVDDVITLPERYDFYIVSQCVRQGTVAPTSYNVIEDSMGLPPEKLQYLT
YKLTHMYYNWSGTVRVPAPCQYAHKLAFMVSQYIHRPAHHDLDNVLYYL

> Ce-Alg1-F48F7.1

MSGGPQYLPGVMNSTIQQQPQSATSSFLPSGPISSTSTSSQVVPTSGATQQPPFPSAQAA
ASTALQNDLEEIFNSPPTQPQTFSDVPQRQAGSLAPGVPIGNTSVSIGEPANTLGGGLPG
GAPGQLPGGNQSGIQFQCPRRPNHGVEGRSILLRANHFAVRIPGGTIQHYQVDVTPDKCP
RRVNREIISCLISAFSKYFTNIRPVYDGKRNMYTREPLPIGRERMDFDVTLPGDSAVERQ
FSVSLKWVGQVSLSTLEDAMEGRVRQVPFEAVQAMDVILRHLPSLKYTPVGRSFFSPPVP
NASGVMAGSCPPQASGAVAGGAHSAGQYHAESKLGGGREVWFGFHQSVRPSQWKMMLNID
VSATAFYRSMPVIEFIAEVLELPVQALAERRALSDAQRVKFTKEIRGLKIEITHCGQMRR
KYRVCNVTRRPAQTQTFPLQLETGQTIECTVAKYFYDKYRIQLKYPHLPCLQVGQEQKHT
YLPPEVCNIVPGQRCIKKLTDVQTSTMIKATARSAPEREREISNLVRKAEFSADPFAHEF
GITINPAMTEVKGRVLSAPKLLYGGRTRATALPNQGVWDMRGKQFHTGIDVRVWAIACFA
QQQHVKENDLRMFTNQLQRISNDAGMPIVGNPCFCKYAVGVEQVEPMFKYLKQNYSGIQL
VVVVLPGKTPVYAEVKRVGDTVLGIATQCVQAKNAIRTTPQTLSNLCLKMNVKLGGVNSI
LLPNVRPRIFNEPVIFFGCDITHPPAGDSRKPSIAAVVGSMDAHPSRYAATVRVQQHRQE
IISDLTYMVRELLVQFYRNTRFKPARIVVYRDGVSEGQFFNVLQYELRAIREACMMLERG
YQPGITFIAVQKRHHTRLFAVDKKDQVGKAYNIPPGTTVDVGITHPTEFDFYLCSHAGIQ
GTSRPSHYHVLWDDNNLTADELQQLTYQMCHTYVRCTRSVSIPAPAYYAHLVAFRARYHL
VDREHDSGEGSQPSGTSEDTTLSNMARAVQVHPDANNVMYFA

> Ce-Alg2-T07D3.7

MFPLPVHNGPRLGKLSIFEMPGDSLTSSSFMPDGGAETSSSSQLGGSAHGAIGTKPDAGV
QFQCPVRPNHGVEGRSILLRANHFAVRIPGGSVQHYQIDVFPDKCPRRVNREVIGCLISS
FSKYFTNIRPVYDGKRNMYTREPLPIGTEPMNFEVTLPGDSAVERKFSVTMKWIGQVCLS
ALDDAMEGRVRQVPHEAVQSIDVILRHLPSLKYTPVGRSFFTPPGVMKPGMQMHQESKLG
GGREVWFGFHQSVRPSQWKMMLNIDVSATAFYRAMPVIEFVAEVLELPVQALAERRALSD
AQRVKFTKEIRGLKIEITHCGAVRRKYRVCNVTRRPAQTQTFPLQLETGQTIECTVAKYF
FDKYRIQLKYPHLPCLQVGQEQKHTYLPPEVCDIVPGQRCLKKLTDVQTSTMIKATARSA
PEREREICKLVSKAELSADPFAHEFGITINPAMTEVKGRVLSAPKLLYGGRHRATTALPN
QGVWDMRGKQFHTGMEVRTWAIACFAQQSHVKENDLRMFTTQLQRISTDAGMPIIGTPMF
CKYASGVEQVEPMFKYLKQTYSAIQLIVVVLPGKTPIYAEVKRVGDTVLGIATQCVQAKN
AIRTTPQTLSNLCLKMNVKLGGVNSILLPNVRPRIFNEPVIFLGCDITHPAAGDTRKPSI
AAVVGSMDAHPSRYAATVRVQQHRQEIITDLTYMVRELLVQFYRNTRFKPARIVVYRDGV
SEGQLFNVLQYELRAIREACVMLESGYQPGITFIAVQKRHHTRLFAADKADQVGKAFNIP
PGTTVDVGITHPTEFDFFLCSHAGIQGTSRPSHYHVLWDDNDLTADELQQLTYQMCHTYV
RCTRSVSIPAPAYYAHLVAFRARYHLVDRDHGSGEEGSQPSGTSSEDTTLSSMAKAVQVH
PDSNNVMYFA

> Ce-Rde1-K08H10.7

MSSNFPELEKGFYRHSLDPEMKWLARPTGKCDGKFYEKKVLLLVNWFKFSSKIYDREYYE
YEVKMTKEVLNRKPGKPFPKKTEIPIPDRAKLFWQHLRHEKKQTDFILEDYVFDEKDTVY
SVCRLNTVTSKMLVSEKVVKKDSEKKDEKDLEKKILYTMILTYRKKFHLNFSRENPEKDE
EANRSYKFLKNVMTQKVRYAPFVNEEIKVQFAKNFVYDNNSILRVPESFHDPNRFEQSLE
VAPRIEAWFGIYIGIKELFDGEPVLNFAIVDKLFYNAPKMSLLDYLLLIVDPQSCNDDVR
KDLKTKLMAGKMTIRQAARPRIRQLLENLKLKCAEVWDNEMSRLTERHLTFLDLCEENSL
VYKVTGKSDRGRNAKKYDTTLFKIYEENKKFIEFPHLPLVKVKSGAKEYAVPMEHLEVHE
KPQRYKNRIDLVMQDKFLKRATRKPHDYKENTLKMLKELDFSSEELNFVERFGLCSKLQM
IECPGKVLKEPMLVNSVNEQIKMTPVIRGFQEKQLNVVPEKELCCAVFVVNETAGNPCLE
ENDVVKFYTELIGGCKFRGIRIGANENRGAQSIMYDATKNEYAFYKNCTLNTGIGRFEIA
ATEAKNMFERLPDKEQKVLMFIIISKRQLNAYGFVKHYCDHTIGVANQHITSETVTKALA
SLRHEKGSKRIFYQIALKINAKLGGINQELDWSEIAEISPEEKERRKTMPLTMYVGIDVT
HPTSYSGIDYSIAAVVASINPGGTIYRNMIVTQEECRPGERAVAHGRERTDILEAKFVKL
LREFAENNDNRAPAHIVVYRDGVSDSEMLRVSHDELRSLKSEVKQFMSERDGEDPEPKYT
FIVIQKRHNTRLLRRMEKDKPVVNKDLTPAETDVAVAAVKQWEEDMKESKETGIVNPSSG
TTVDKLIVSKYKFDFFLASHHGVLGTSRPGHYTVMYDDKGMSQDEVYKMTYGLAFLSARC
RKPISLPVPVHYAHLSCEKAKELYRTYKEHYIGDYAQPRTRHEMEHFLQTNVKYPGMSFA

> Ce-PPW1-C18E3.7

MEKQLEAMFVSDRPAAPAAQKLGTAPLAAKKTRNVERGTKVNIDTNIRKLTIKPNQPIYK
YAVQVNYVFRKPDGTEATIEMSKSAKKGTEHDNDKTRCQKVYNEAIKRYDELKTGGPFFY
DRQASLYTLTKLKNESISFVVTDKICKRQNFKEAQFVLKKVDQSFQSTSNDVIRTTNSCP
ANADKTLLEAMNIIVSGPAFENKNVITVGACVHYLIDPTVVDVAYKEYAEGQLYSGVGAS
KSVKTLEGTDKKVPSLFMTTEMKTTLFHPDYAPLVELLQTFRGFSTTLKANSPAAQRIEK
AFVGLDVVLNYGVHKGLGEDGVVMKIRRFHTSAKETCFEVEKSTREFTNVFDYFKKKYGI
TLKYPDLFTIEAKGKQGKIHFPAEVLLLCPNQTVTNDQMINNEQADMIKMSAAQPHIRKT
TTDTIVRNVGLASNNIYGFIKVEDPVNLEGMVLPKPKIAFAGNQLADLANPKSRFPTDFN
RAGQYYDAKELTKWELVFVQNEEVQGLAKQLADEMVNNGMKCSNPTMSFIIRGDLEPIFK
KAKAAGTQLLFFVVKSRYNYHQQIKALEQKYDVLTQEIRAETAEKVFRQPQTRLNIINKT
NMKLGGLNYAIGSEAFNKPNRLIVGFVTSQRVGGNPDYPISVGFAANMLKHHQKFAGGYV
YVHRDRDVFGSIIKDTLLAIFKTCTEQRGRPDDILLYFNGVSEGQFSMINEEFSARVKEA
CMAFQKEGTPPFRPHITIIASSKAHNERLYKSDKGRIVNLEPGTVVDHTIVSNVYTEWYH
ASAVARQGTAKATKFTLIFTTKAGPQAEPLWHLEQLTNDLCYDHQIVFHPVGLPVPLYIA
DRYSQRGAMVLAANQGPIYNEGQIDLAATNSAYGYGEKKLFTTRFNA

> Ce-PPW2-Y110A7A.18

MPATPVPPVTMPPVPPVGFPPVTAPPGLHPPPPVPPVPVPTLPVTSEHKTAHDACIKRLE
QLEIPPDPKIYPSPTEPGTFGSVTEVLTNVFGVEVHKTSNFYQYSVHITAELSSKKEVTF
TKKGKEDFIVHDRHEKCSAILFHALNRFEDFFKSSENCIIYDGQSILFTSMNLFEGIPTG
KIKTKVFQINGADTNLKDLMVLPCIKLEVFPTKNPVVNFSLEDIGRRTSDSNIESVNMAY
KQILDLAMSQYAIRETSKCVVFEHGKMFFINPLQEGYAPCDIVEVGDGKQMKPGLKKTLQ
FIEGPYGRNQNNPSIVIDGMKVAFHMEQLVFQKLSSITSNLANGITGSERERCAAVIKGL
DCHSNYRGRTRHHKIESIHHEGAATARFELEGGGTCTVATYFKDKYKIQLRYPNANLIVC
KERGALNFYPMELITISPNQRVRITQQTSSQSQRTTKESAVLPDIRQRLIMTGKIAAKIT
AENEVLGKMGVTVCDEPLVVKGRNLPAIRLASFETGEHLINPRDCKWRPQRYNRSAVAPK
VWALYGVGSPGSQMNRDVMRRFCDEFMNMSRSKGILFPPPGDVNLLTPDAIENRLREAAN
AGCTFVLCITEDNITCLHQKYKFIEHHTQMIVQDMKLSKALSVVNASKKLTLENVINKTN
VKLGGSNYVYLDTKNFLQEHLIIGVGISSPPPGTKYIMESRGILNPTIVGFAYNGNGKQE
FSGDFVLNAAGQETIAPIEDIVSYSIKGYKKFHDGKAPKRITIYRSGSSEGNHGPIISYE
VPLARVAMRNFSPDTQLLYIVVSKEHTYRFFKKESGGSSSGGSNSAGASNSGTLTSAPPK
PWELNIGPGLTVDYGVTNPACKQFFLNSHMTLQGSAKTPLYTVLADDRNIGMSALEEFTF
NLCHLHQIVGLPTSIPTPLYVANEYAKRGRNLWNEAINANEIPEVTGPESARLKKMTDGI
SYMGSGDMIDRRVNA

> Ce-Sago1-K12B6.1

MSNITQVTSSMASASLSNKAPLPVGHQPLAEKKPKEVNQEGTPVQIVTNMRKINLEKNHS
IFKYSVQVLFVYQKSDGTELVLEKSKSVGSGCDHERSKSHCLRVYRKAAKQCQELKSGGP
FCYDSQGCLYSFSKLKNDEFSTNITGSDISNNPKFLRVEFKLAKVQESFQTTTNDVAKSV
NCRPALQEKTILEAMNQIVSTAPINHPNVLTIGNCVHYLYDDTNIDIRSITGEGGKSSAV
GASKSVRTLEGTGKTPCLYMATELKTTLFHPDNCSLLKVFMDYRGFNGSLKANSPFVLKN
KNAFIGLWCYTTHGKCSDWKDDRPMIKIKDFGLSAKETTFERDNKKISVFNYFQVKYNMT
LKYPDLFTVVARGKDGKNQHIPVECLDLCNSQTVRTEQMVGTEQADLIKLAAAKPHDRKK
ITDTVVNSIGLASEPKGIISVGAPESVTGLVLPKPDIYFSGGKKVFWNDPKKRGPATDFM
PAGTFIKPTKLTNWEVVFDNGVQLVDCIQHLTSTMRQLGMEVSNPTVSLINRGYLRSIFE
NAKAANRQLIMFITKSMNNYHTEIKCLEQEFDLLTQDIRFETAVKLAQQQNTRKNIIYKT
NMKLGGLNYELRSGVFSNSKRLIIGFETSQRGGLGDAPIAIGFAANMMSHSQQFAGGYMF
VKKSADNYGPVIPEILLTILKQAKANRPNDRPDELLIYFSGVSEGQHALVNEYYANQVKA
ACGLFNESFRPHITLILASKVHNTRVYKSENGGGVCNVEPGTVIDHTIVSPVLSEWYHAG
SLARQGTSKLVKYSLIFNTKKNEKLSVYERLTNELCYEMQIVFHPTSLPIPLHIAGTYSE
RGSQMLALKKPIYTNGEFNQVATNEQLGYASKKLFGTRFNA

> Ce-Sago2-F56A6.1

MEKQLKAMSVSDKPAAPAAQKLGTAPLAAKKTRNEEWGTKVNIDTNIRKLTIKPNQPIYK
YAVQVNYVFRKPDGTEATIEMSKSAKKGTEHDNDKTRCQNVYNEAIKRYDELKTGGPFFY
DRQASLYTLTKLKNESISFVVTDKICKRQNFKEAQFVLKKVDQSFQSTSNDVIKTTNSCP
ANADKTLLEAMNIIVSGPAFENKNVITVGACVHYLIDPTGVDVAYKEYPEGQLYSGVGVS
KSVKTLEGTDKKVPSLFMTTEMKTTLFHPDYAPLVELLQTFRGFSTTLKANSPAAQRIEK
AFVGLDVVLNYGVHKGLGEDGVVMKIRRFHTSAKETCFEVEKSTREFTNVFDYFKKKYGI
TLKYPDLFTIEAKGKQGKIHFPAEVLLLCPNQTVTNDQMINNEQADMIKMSAAQPHIRKT
TTDTIVRNVGLASNNIYGFIKVEDPVNLEGMVLPKPKIAFAGNRLADLANPKSRFPTDFN
RAGQYYDAKELTKWELVFVQNEEVQGLAKQLADEMVNNGMKCSNPTMSFIIRGDLEPIFK
KAKAAGTQLLFFVVKSRYNYHQQIKALEQKYDVLTQEIRAETAEKVFRQPQTRLNIINKT
NMKLGGLNYAIGSEAFNKPNRLIVGFVTSQRVGGNPDYPISVGFAANMLKHHQKFAGGYV
YVHRDRDVFGSIIKDTLLTIFKTCTEQRGRPDDILLYFNGVSEGQFSMINEEFSARVKEA
CMAFQKEGTPPFRPHITIIASSKAHNERLYKSDKGRIVNLEPGTVVDHTIVSNVYTEWYH
ASAVARQGTAKATKFTLIFTTKAGPQAEPLWHLEQLTNDLCYDHQIVFHPVGLPVPLYIA
DRYSQRGAMVLAANQGPIYNEGQIDLAATNSAYGYGEKKLFTTRFNA

**Table S4:** Accession numbers of RNA-dependent RNA polymerase (RdRP) protein sequences used in phylogenetic tree construction.

| **Species Name** | **Abbreviation of RdRP proteins used in tree construction** | **Accession numbers** | **Database** |
| --- | --- | --- | --- |
| ***Metaseiulus occidentalis* (Mo)** | Mo1 | XP_028966489.1 | NCBI |
|  | Mo2 | XP_028967682.1 | NCBI |
|  | Mo3 | XP_028966390.1 | NCBI |
|  |  |  |  |
|  |  |  |  |
| ***Varroa destructor* (Vd)** | Vd1 | XP_022658093.1 | NCBI |
|  | Vd2 | XP_022666953.1 | NCBI |
|  | Vd3 | XP_022666954.1 | NCBI |
|  | Vd3 | XP_022647785.1 | NCBI |
|  |  |  |  |
| ***Varroa jacobsoni* (Vj)** | Vj1 | XP_022702066.1 | NCBI |
|  | Vj2 | XP_022702067.1 | NCBI |
|  | Vj3 | XP_022702068.1 | NCBI |
|  | Vj4 | XP_022702069.1 | NCBI |
|  | Vj5 | XP_022702071.1 | NCBI |
|  |  |  |  |
| ***Ixodes scapularis* (Is)** | Is1 | XP_029828638.1 | NCBI |
|  | Is2 | XP_029830748.1 | NCBI |
|  | Is3 | XP_029847912.1 | NCBI |
|  | Is4 | XP_029848105.1 | NCBI |
|  |  |  |  |
| ***Tropilaelaps mercedesae* (Tm)** | Tm1 | OQR78246.1 | NCBI |
|  | Tm2 | OQR80325.1 | NCBI |
|  | Tm3 | OQR79267.1 | NCBI |
|  |  |  |  |
| ***Tetranychus urticae* (Tu)** | Tu1 | XP_015794858.1 | NCBI |
|  | Tu2 | XP_015794859.1 | NCBI |
|  | Tu3 | XP_015794860.1 | NCBI |
|  | Tu4 | XP_015794862.1 | NCBI |
|  | Tu5 | XP_025018610.1 | NCBI |
|  |  |  |  |
| ***Dermatophagoides pteronyssinus* (Dp)** | Dp1 | XP_027197271.1 | NCBI |
|  | Dp2 | XP_027203264.1 | NCBI |
|  | Dp3 | XP_027203039.1 | NCBI |
|  | Dp4 | XP_027200645.1 | NCBI |
|  | Dp5 | XP_027200646.1 | NCBI |
|  |  |  |  |
| ***Euroglyphus maynei* (Em)** | Em1 | OTF70706.1 | NCBI |
|  | Em2 | OTF80411.1 | NCBI |
|  | Em3 | OTF77947.1 | NCBI |
|  | Em4 | OTF75937.1 | NCBI |
|  |  |  |  |
| ***Sarcoptes scabiei* (Ss)** | Ss1 | KPM11269.1 | NCBI |
|  |  |  |  |
| ***Psoroptes ovis* (Po)** | Po1 | SZF06453.1 | NCBI |
|  | Po2 | SZF06468.1 | NCBI |
|  | Po3 | SZF06521.1 | NCBI |
|  |  |  |  |
| ***Caenorhabditis elegans* (Ce)** | Ce˗ego1 | F26A3.3* | WormBase |
|  | Ce˗Rrf1 | F26A3.8* | WormBase |

*- The amino acid sequences of the query proteins are presented below and those of the RdRP domain (IPR007855/PF05183) retrieved from the Pfam database appear in red within the sequence of the query proteins.

> Ce˗ego1- F26A3.3

MGDEGYRGWIKLEIPCSLPERQMGPIVKCHVAKLEPALNEYNIKVLTKGQVQVVEEQDCE
PFYETNYEVATSRFSHDLIAAIQTYLKDLSTDHLMPFQRGNLVLHSSDFWSSELTCHLVD
IPLAAVFFGNIQGGTFINHWEVSFWDDVRRRKSARTRNTEPTQADKIGMNQIKVEFEFDK
IDFMTVHFKHFENDFEVADKDAKRTKQTVTMYYQITVRRTSIRRIIVDPVVQDCNGSDRI
RVHFELNCPVLIRRAYRTAKQESENRHSVPHYRRYLVINRGRSANQYPTAKAITDSPVFT
IEFDQSVGLNEIYRLLSRLRIRTGVSIEFADIPSIDCLIWRENPYNRWTFLNNQHLSPTH
FSAPIYRDFITTAFPKKHEVCGSREVDTNRERKFAITYLLECLISRGAVVKDQILLDEGI
WHRFLEVILHYYTKDDKLCEAGLEDLVHMIDGRKRIGSLIKCFDRICQTRQRNSLVNGLT
TEEMREGYQRVRKIIFTPTRVIYVAPETLMGNRVLRRYDHDGTRVLRITFRDDDNQKMRT
NKTSTMLEKTVNQYLKNGITVAGRNFGYLGSSNSQMRDNGAYFMEKYSSSQCREYERIYQ
IKPPITFNPKIQAARKNLGRFETIDNIPKMMARLGQCFTQSRLSGVNLERCTYMTTYDLT
GGKNLKGDEYTFSDGVGMMSYRFAQMVSEVMDFGKGVPSCFQFRFRGMKGVISIEPLLDN
LRQWSISYNISKPSDDSSWSLNCMFRPSQIKFISKRHPRDQVEIVKYSSPVPVALNKPFI
NILDQVSEMQSLECHRRVTNRIEELLDRQMLSFAQQMVDETFCRNRLKELPRRVDIDYLR
TTWGFTLSSEPFFRSLIKASIKFSITRQLRKEQIPIPCDLGRSMLGVVDETGRLQYGQIF
VQYTKNLALKLPPKNAARQVLTGTVLLTKNPCIVAGDVRIFEAVDIPELHHMCDVVVFPQ
HGPRPHPDEMAGSDLDGDEYSIIWDQQLLLDKNEDPYDFTSEKQKASFKEDEIDDLMREF
YVKYLKLDSVGQISNSHLHNSDQYGLNARVCMDLAKKNCQAVDFTKSGQPPDELERKWRK
DEETGEMIPPERAERVPDYHMGNDHTPMYVSPRLCGKLFREFKAIDDVLKISEERDEQVE
ISIDETIKIDGYTEYMASAKNDLARYNAQLRSMMENYGIKTEGEVFSGCIVDMRNRISDK
DQDDMSFFNTNQMIETKLTNLFKKYREIFFEEFEGGWEGNTEAFSRYGRDSNILQRQCRA
PTVQMMKKAVAWYRACYEEARITRENKKLSFAWLAYDVIAKVKQDKSLTSDEVKMGGANP
LYTMLDDHRSQYLVDNSRKFEAFRQFSTPKTSGEQVKRAHRIIKMYTETYPGLDAVLFML
DEWARISNLFENQSLREYHLSLLFILFATRQFSSVDGNAAKFFNKVDEKSYKQSKTIGDF
EPSLYIEEKGKSQMMVKFLEFLASRKFRKMANLSFCALDFSSIFMRGEWQIFHLAALKTY
YNVLFNLRFEELPVSTDPTTTVRSIIRENEPFVIELPANCDRSLVHRKLVEHTGVKEIFM
RNMEKSVRSSDDVQKINMRLLVSTRGTLESMYKLRQLVAVKVPIKTYVTGQDVSTQMARL
CYEKIVRGHINI

> Ce˗Rrf1-F26A3.8

MSERSHGFIKFEFPEFDNSLQVIEGSIDTMLLTFTSVLRKYEIEIKSRQETQIVEEQDCD
CFFEINFEVESEQFDHTIIDAMHDYLSDLNVYVPYQRPNIVLHGSDFWLRTLDCHTEIPL
AAIYFGNIQGGTYFNHWQVSFSRENISSRDMLHKIHAEFEFDKTDMITVQFQCFEEKKQK
FEDSRKQKVRVNYQLTIRRDSIRRIIVDPRVEGCNTCVHFEVNCPPLIRKGYIDNDKSSF
HKPFYERQKRFDCDWRNGNVNHGNPQDAAIADSPFFTIEFHKEISTKEMYRVLSRLRSRT
KVLIEFANLPSIDVPMGSHYPYNRWNLKKSPTDSNAPIFREFLKEIFPPKYEIVDDKLID
VNEERKFSITYLIECLLSRGAIVKDQLLLNEQHWKNFLEIIIWYYRNDNQLCEAALEDLV
HLIDGRKRIGSILKCLDKICQKREVMKLVNGLTEKESIEGYQRVRKVIFTPTRVIYIAPE
TIMGNRVLRKFDKDGTRVLRVTFRDDNNKKMRSNVTGKLLDRTANKYLEHGVRIANREYG
FLGCSNSQMRDNGAYFMMRFTDKQLDRFYKCNPTASNINFKPKIDEVRFQLGRFSEIENV
PKLMARLGQCFTQSRLTGVGLGRDDYCSTYDLTGGRATNGSEYTFSDGVGMMSYQFAQEV
SQAMQFGKAVPSCFQIRFRGNKGVIAIEPFLDEIRKWALVNGVTSMKMAKCLFRPSQIKF
QAKAISGDQIEMVKFSSAVLVALNKPFINILDQVSEMQSLDCHKRITSRIEELMDRQILS
FAKQMNEETFCRNKLKEFPRRIDIDNLRTMWGFTLSSEPFFRSLIKASIKFSITKQLCKE
QIQIPSELGRSMLGVVDETGRLQYGQIFVQYTKNYKKKLPPRDSNNKVHGSEIVTGTVLL
TKNPCIVPGDVRIFEAVDIPELHHMCDVVVFPQHGPRPHPDEMAGSDLDGDEYSVIWDQE
LLLERNEEPFDFAVEKIKVPYDREKLDVLMREFYVTYLKLDSVGQISNSHLHNSDQYGLN
SRVCMDLAKKNCQAVDFTKSGQPPDPLETKWRADPVTFEVIPPENPERIPDFHMGNERSP
MYVSPRLCGKLFREFQAIDNVIKISEERDEQYNIELDETIFVTGFERYMESAQKQLSSYN
GQLRSIMENYGIRSEGEIMSGCIVEMRNRISDKDQDDMSFYNTNQMIETKMTSLVCKFRE
TFFEEFGGFTVKCTLLPNAYDNGNCLNYRCEDPDQEVRKKAVAWYRACYECAQSTREVRK
LSFAWIAYDVIAKVKETNVLNNERMQIGGANPMYTFLEEHRKQYLIDHDADFKNFCELDH
LITGEKSKEAISILKIYLEMIPGLDSVFFMLMRWGESLRLFDGKPIKIYHFFLMFILFAT
RQLASADGNAEPFFKIIEKEEYEKQKRDSSRGNIDPLTEKKRSDMMVKFFQFMGCRKFRK
MSTLSFCPLNFSSIFMRGEWRIFHESALKTYYNILFNLRFEELPVSSDPTITAETMDREC
EPFVIELPENINVNDLINNMKKHTNVSTVKMRRQEKNPINDKAKPKTTVRYIVSVSGTLE
SIQMLKKLSAVTIPIKSHWEGEEVSQQMASLCYQKVMNGEF
